# The Cellular and Viral circRNAome Induced by Respiratory Syncytial Virus Infection

**DOI:** 10.1128/mBio.03075-21

**Published:** 2021-12-07

**Authors:** Wenxia Yao, Jinghui Pan, Zhaoyu Liu, Zhijie Dong, Min Liang, Shu Xia, Yao Xiao, Xiaodan Cai, Tao Peng, Xinke Zhou, Hua Cai

**Affiliations:** a The Fifth Affiliated Hospital of Guangzhou Medical University grid.410737.6Guangzhou Medical University, Guangzhou Medical University, Guangzhou, China; b Guangzhou Hoffmann Institute of Immunology, College of Basic Sciences, Guangzhou Medical University, Guangzhou, China; c The First Affiliated Hospital of Guangzhou Medical University grid.410737.6Guangzhou Medical University, Guangzhou Medical University, Guangzhou, China; d Guangzhou Gene Denovo Biotechnology Co. Ltd., Guangzhou, China; Tulane University Health Sciences Center and Tulane Cancer Center; Brown University

**Keywords:** RSV, cellular circRNAs, viral circRNAs, antiviral immune response

## Abstract

Circular RNAs (circRNAs) are a new class of noncoding RNAs that have gained increased attention. DNA virus infections have been reported to induce modifications in cellular circRNA transcriptomes and express viral circRNAs. However, the identification and expression of cellular and viral circRNAs are unknown in the context of respiratory syncytial virus (RSV), a human RNA virus with no effective treatments or vaccines. Here, we report a comprehensive identification of the cellular and viral circRNAs induced by RSV infection in A549 cells with high-throughput sequencing. In total, 53,719 cellular circRNAs and 2,280 differentially expressed cellular circRNAs were identified. Trend analysis further identified three significant expression pattern clusters, which were related to the antiviral immune response according to gene enrichment analysis. Subsequent results showed that not only RSV infection but also poly(I·C) treatment and another RNA virus infection induced the upregulation of the top 10 circRNAs from the focused cluster. The top 10 circRNAs generally inhibit RSV replication in turn. Moreover, 1,254 viral circRNAs were identified by the same circRNA sequencing. The induced expression of viral circRNAs by RSV infection was found not only in A549 cells but also in HEp-2 cells. Additionally, we profiled the general characteristics of both cellular and viral circRNAs such as back-splicing signals, etc. Collectively, RSV infection induced the differential expression of cellular circRNAs, some of which affected RSV infection, and RSV also expressed viral circRNAs. Our study reveals novel layers of host-RSV interactions and identifies cellular or viral circRNAs that may be novel therapeutic targets or biomarkers.

## INTRODUCTION

Respiratory syncytial virus (RSV) is a nonsegmented RNA virus that belongs to the genus *Pneumovirus* of the family *Paramyxoviridae* (1). Similar to other negative-strand RNA viruses, the RSV genome is a template for two processes, namely, transcription that yields mRNAs and replication that yields an antigenome RNA; the antigenome, in turn, acts as a template for genome synthesis. RSV infection occurs in over 95% of humans by 2 years of age and recurs throughout life. RSV infection can result in serious respiratory symptoms in infants and the elderly, and it is one of the leading causes of infant mortality worldwide (second only to malaria) (2). Treatment strategies for RSV infection are limited to palivizumab, which is a costly monoclonal antibody that is approved only for prophylaxis in high-risk infants, and ribavirin, which has limited efficacy and entails serious safety concerns (3). Despite substantial effort, including the preclinical or clinical development of various vaccine candidates, a safe, effective, clinically approved RSV vaccine has not been developed to date (4, 5).

Eukaryotic cells produce several classes of noncoding RNAs (ncRNAs), and circular RNAs (circRNAs) are a newly identified class of ncRNAs that have recently elicited increased attention (6–8). circRNAs are formed by the back-splicing of a downstream splice donor site to an upstream splice acceptor site, thus producing a covalently closed RNA molecule with no 5′ caps or 3′ poly(A) tails. circRNAs are unusually stable, presumably because their lack of free ends is resistant to exonuclease activity, which enables circRNAs to serve as a new class of potential biomarkers. Although our understanding of the functions of circRNAs is still nascent, an increasing number of studies have shown that circRNAs can sequester microRNAs or proteins, modulate transcription, interfere with splicing, and even translate to produce polypeptides or proteins (9). Furthermore, emerging lines of studies have revealed that some circRNAs play important roles under physiological and pathological conditions, and they may serve as diagnostic or predictive biomarkers of diseases and provide new potential therapeutic targets (10).

Viral infections can induce modifications in cell transcriptomes, including the circRNA transcriptome. Shi et al. and Tagawa et al. have respectively reported that infections by the human DNA viruses herpes simplex virus (HSV) and Kaposi’s sarcoma-associated herpesvirus (KSHV) induce changes in cellular circRNA transcriptomes (11, 12). As virus hosts, eukaryotic cells rely on their innate immune response as the first line of defense against viruses. Similar to their host cells, many viruses synthesize their own viral ncRNAs, and multiple biological roles, including the regulation of viral replication or persistence and host immune evasion, have been ascribed to viral ncRNAs (13). Several research teams discovered and validated the presence of viral circRNAs in DNA viruses (14, 15) such as Epstein-Barr virus (EBV) and KSHV with large genomes (16–18) and human papillomaviruses (HPVs) with small genomes (19). In addition, Cai et al. predicted some circRNAs in RNA viruses by systematic bioinformatics analysis of the viral infection-related RNA sequencing (RNA-seq) data (20).

However, the identification and characterization of cellular and viral circRNAs are unknown in the context of RSV, a human RNA virus with no effective treatments or vaccines: whether or not the expressions of cellular circRNAs are regulated by RSV infection and subsequently affect RSV infection and whether there are viral circRNAs derived from RSV remain unclear. In the present study, we performed a comprehensive identification and expression analysis of cellular and viral circRNAs induced by RSV infection in A549 cells. We revealed novel cell-virus interactions of circRNAs: cellular circRNAs were induced in response to RSV infection and potentially affected RSV infection, and RSV could also express viral circRNAs.

## RESULTS

### Identification and profiling of cellular circRNAs in mock- and RSV-infected A549 cells.

**FIG 1:**
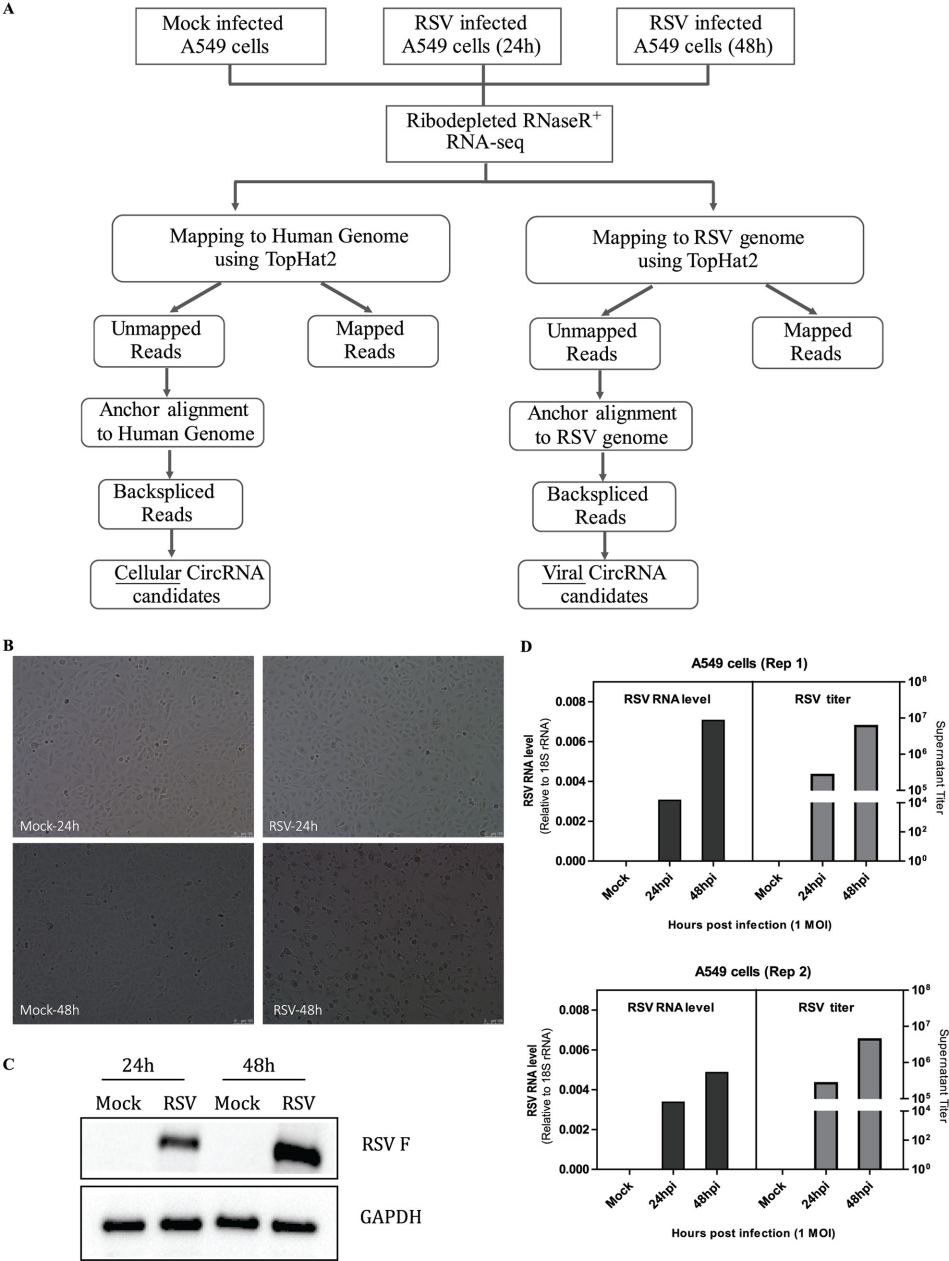
Experimental schema for the identification of cellular and viral circRNA candidates in mock- and RSV-infected A549 cells and characterization of mock and RSV infection in A549 cells. (A) Flowchart demonstrating how the high-throughput sequencing data were analyzed and how cellular and viral circRNAs were identified. Briefly, each sample RNA was ribodepleted and treated with RNase R prior to library preparation, and each sample was sequenced using an Illumina HiSeq 2500 instrument. The obtained reads were mapped to the human genome and the RSV genome by TopHat2. Next, 20-mers from both ends of the unmapped reads were extracted and aligned with the human or RSV genome to determine unique anchor positions. Anchor reads that aligned in the reverse orientation indicated circRNA back-splicing and were subjected to find_circ to identify cellular or viral circRNAs. A candidate circRNA was called when it was supported by at least two back-splicing reads. Each group has two repetitive samples. (B to D) Characterization of RSV infection in A549 cells at 24 and 48 h, with mock infection as a negative group. (B) Induction of cytopathic effects in A549 cells. (C) Analysis of RSV F protein expression by Western blotting. (D) Analysis of intracellular RSV RNA levels by qRT-PCR and extracellular RSV titers by plaque assays in two repetitive samples. In panels A to D, the multiplicity of infection (MOI) was 1.

To characterize circRNA transcripts, we performed RNA sequencing (RNA-seq) analysis of rRNA-depleted and RNase R-treated total RNA isolated from A549 cells infected with RSV at 24 and 48 h, with mock-infected A549 cells as a negative group (Fig. 1A). The multiplicity of infection (MOI) was 1, and each group had two repetitive samples. As shown, RSV-infected A549 cells showed an apparent cytopathic effect, especially at 48 h (Fig. 1B), and RSV F protein expression (Fig. 1C); moreover, RSV-infected A549 cells demonstrated robust RSV RNA expression and virus production (Fig. 1D). Each sample was sequenced on an Illumina HiSeq 2500 instrument, and >15 billion bases of data were produced. The obtained reads, which were filtered residual rRNA reads, were mapped to the human reference genome (GRCh38) and the RSV genome (GenBank nucleotide sequence accession no. M74568.1) by TopHat2 (Fig. 1A). Next, the unmapped reads were extracted, and a computational pipeline based on anchor alignment was implemented to identify cellular and viral circRNAs (Fig. 1A). Briefly, 20-mers from both ends of the unmapped reads were extracted and aligned to the reference human or RSV genome to determine unique anchor positions. Anchor reads that aligned in the reverse orientation indicated circRNA back-splicing and were then subjected to find_circ (6) to identify circRNAs (Fig. 1A). A candidate circRNA was called when it was supported by at least two unique back-splicing reads. A detailed summary of the RNA-seq data sets for each sample is provided in Table 1.

**TABLE 1:** Summary of RNA-seq data sets from mock- and RSV-infected A549 cells^*a*^

Sample	Total no. of reads	Mapping ratio to human genome (%)	No. of cellular circRNAs	Mapping ratio to RSV genome (%)	No. of viral circRNAs
Mock_*Rep1*	106,966,198	92.35	25,609	0.00	0
RSV-24h_*Rep1*	103,175,460	91.98	25,445	0.67	35
RSV-48h_*Rep1*	106,871,262	86.14	26,455	6.10	906
Mock_*Rep2*	97,770,190	92.55	24,726	0.00	0
RSV-24h_*Rep2*	102,777,188	92.03	25,102	0.39	46
RSV-48h_*Rep2*	104,870,684	89.30	25,523	3.30	688

Total			53,719		1,254

a The human genome was GRCh38; the GenBank nucleotide sequence accession no. of the RSV genome is M74568.1. Mock, mock infected cells. RSV-24h, RSV infected cells (24h). RSV-48h, RSV infected cells (48h). Rep 1, repetitive sample 1. Rep 2, repetition sample 2.

**FIG 2:**
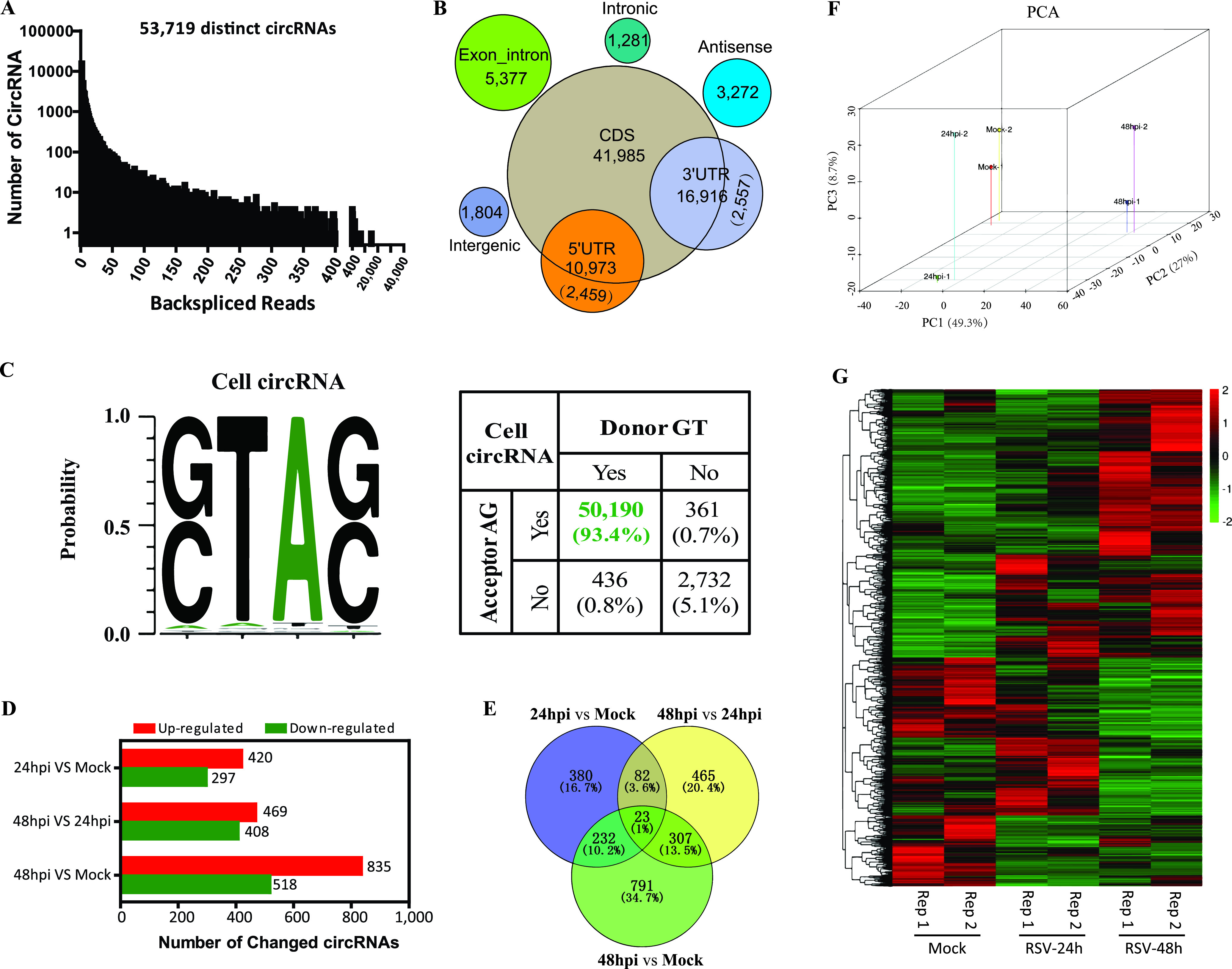
Profiling of cellular circRNAs and characterization of differentially expressed cellular circRNAs in mock- and RSV-infected A549 cells. (A) Numbers of cellular circRNAs and back-spliced reads identified in all samples. (B) Genomic region annotations and numbers of cellular circRNAs. (C) Back-splice signals of cellular circRNAs. (D) Bar plots demonstrating the numbers of differentially regulated circRNAs in each paired group. (E) Venn diagram presenting the number of differentially expressed circRNAs that are unique or common in each paired group. (F) Principal-component analysis (PCA) of the 2,280 differentially expressed cellular circRNAs. (G) Clustered heat map for the 2,280 differentially expressed cellular circRNAs from all samples, with rows representing circRNAs and columns representing samples. The numerical data represent normalized log_10_-transformed RPM.

In total, 53,719 distinct cellular circRNA candidates were identified by this approach (Fig. 2A); 15,700 of them were matched in circBase (21), and 38,019 of them were novel circRNAs. The circRNA distributions of each group on human chromosomes showed that circRNAs were widely distributed among all chromosomes, including sex chromosomes, with the largest number of circRNAs on chromosomes 1 and 2 and the smallest number on chromosome Y (see Fig. S1A and B in the supplemental material). We then annotated the identified circRNA candidates using the RefSeq database (22). Approximately 78% of the circRNAs originated from coding DNA sequences (CDSs), whereas smaller fractions aligned with the 5′ untranslated region (UTR), the 3′ UTR, exon-introns, or antisense sequences (Fig. 2B). The average and median lengths of the cellular circRNAs were 2,386 and 637 nucleotides (nt), respectively (Fig. S1C). The splicing signals of the cellular circRNAs were also investigated, and the results indicated that most (93.4%) of the splicing signals had a canonical GT/AG donor/acceptor sequence (Fig. 2C), which is consistent with the previously reported circRNAs in mammals (23).

10.1128/mBio.03075-21.2 FIG S1 Cellular circRNA distributions of each group on human chromosomes. (A) Overview of all the cellular circRNA distributions from different groups on different chromosomes. (B) Numbers of cellular circRNAs from different groups on different chromosomes. (C) Length distribution of cellular circRNAs. (D) Three volcano plots demonstrating the distributions of differentially regulated circRNAs in each paired group. Significantly upregulated circRNAs are indicated in red, and downregulated circRNAs are indicated in green (FC of ≥2.0 or FC of ≤0.5 and *P* value of <0.05). Download FIG S1, PDF file, 2.1 MB. Copyright © 2021 Yao et al. 2021 Yao et al. https://creativecommons.org/licenses/by/4.0/ This content is distributed under the terms of the Creative Commons Attribution 4.0 International license.

### Characterizing differentially expressed cellular circRNAs in mock- and RSV-infected A549 cells.

circRNA expression in mock- and RSV-infected A549 cells was calculated based on RPM (back-splicing junction reads per million mapped reads), which permits quantitative comparisons. Filtering analysis by the criteria of a fold change (FC) of >2.0 or <0.5 and a *P* value of <0.05 identified 2,947 cellular circRNAs whose expression was differentially regulated among the three groups (Fig. 2D). The distributions and numbers of upregulated or downregulated circRNAs are shown in volcano plots (Fig. S1D) and bar charts (Fig. 2D), respectively. As shown in Fig. 2D, the RSV-infected group at 48 h postinfection (hpi) had the largest number of differentially regulated circRNAs, that is, 835 upregulated and 518 downregulated circRNAs, compared with the mock group; additionally, 469 upregulated and 408 downregulated circRNAs were identified when the RSV 48-hpi group was compared with the RSV 24-hpi group, and 420 upregulated and 297 downregulated circRNAs were identified when the RSV 24-hpi group was compared with the mock group.

Furthermore, a Venn diagram was plotted to display the differently and commonly regulated circRNAs, and the results showed that three groups shared 23 common differentially expressed circRNAs, with each paired group having 82, 232, and 307 commonly regulated circRNAs (Fig. 2E). Combining the data in Fig. 2D with those in Fig. 2E, there were in total 2,280 nonoverlapping differentially expressed circRNAs. Three-dimensional principal-component analysis (PCA) of the 2,280 differentially expressed circRNAs revealed that each of the three groups could be separated from the other two groups by plotting the three largest sources of variance (PC1, PC2, and PC3) (Fig. 2F). Clustering analysis of the 2,280 differentially expressed circRNAs suggested different expression patterns of circRNAs in the three groups (Fig. 2G).

### Identification of temporally expressed clusters with 2,280 differentially expressed circRNAs and further function enrichment analysis.

**FIG 3:**
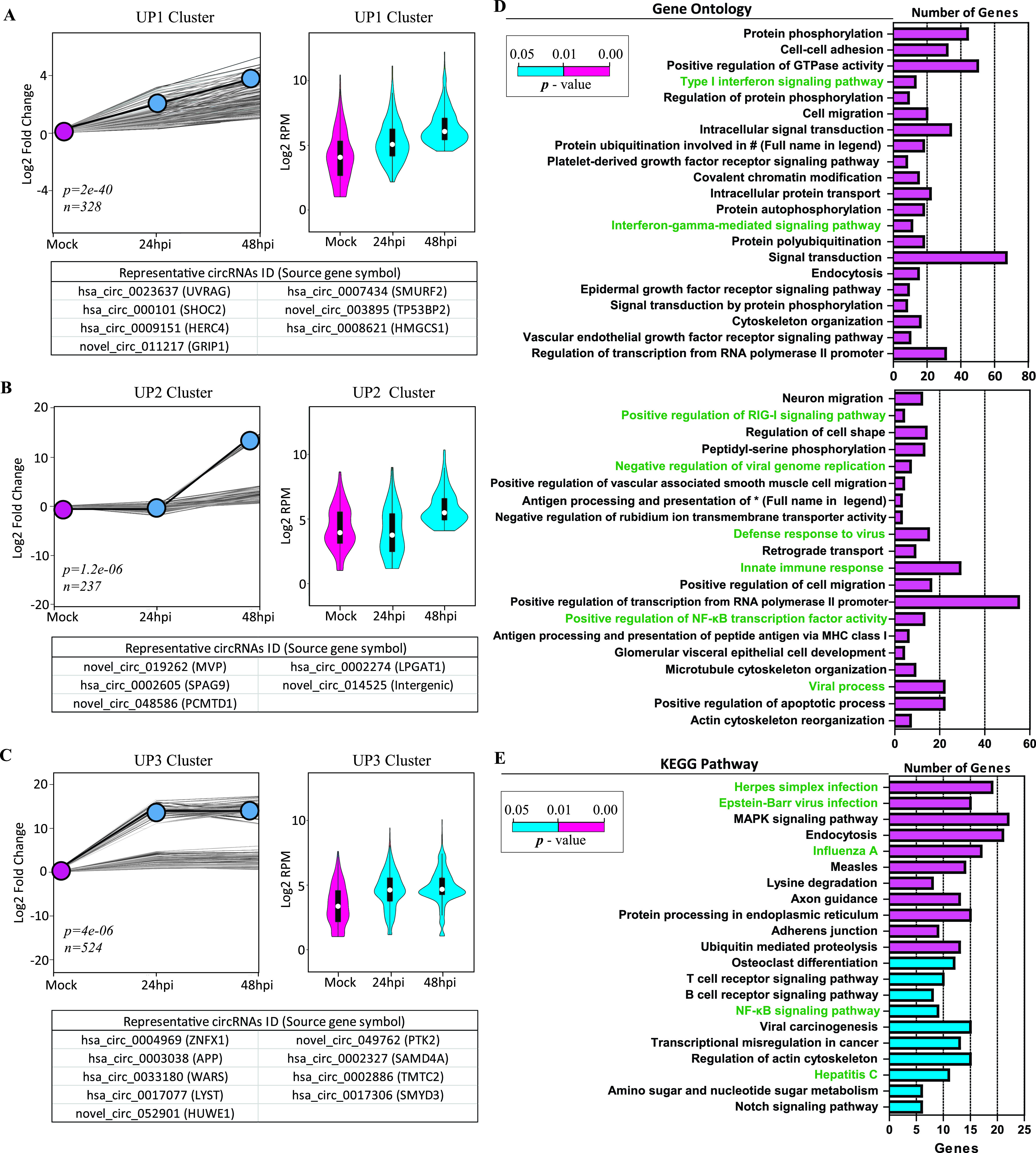
Trend analysis of temporally expressed clusters with 2,280 differentially expressed circRNAs and further GO and KEGG enrichment analyses of circRNAs from the three significant expression clusters. (A to C) Time series analysis of the 2,280 differentially expressed circRNAs with a 2-fold change in at least one time point. Results show three significant temporal clusters by STEM analysis (*P* < 0.05). (Left) Line plots were used to show fold changes (the number of circRNAs assigned to each cluster and *P* values are shown). The gray lines indicate the profiles of individual circRNAs, and the black lines show the model profile for each cluster in the line plots. (Right) Violin plots were used to show the absolute expression levels (log_2_ scale). (Bottom) Representative circRNAs with the source gene symbol in parentheses for each cluster. (D and E) GO (D) and KEGG pathway (E) enrichment analyses for parental protein-coding genes of circRNAs from the three significant temporal expression clusters. Enriched GO terms and KEGG pathways associated with virus infections and the immune response are indicated in green (#, ubiquitin-dependent protein catabolic process). MHC, major histocompatibility complex; MAPK, mitogen-activated protein kinase.

To unveil the dynamically expressed RSV-associated circRNAs, we performed short time series expression miner (STEM) clustering (24). We obtained three significant temporal expression clusters (*P* < 0.05) (Fig. 3A to C) and five nonsignificant temporal expression clusters (Fig. S2) with the 2,280 differentially expressed circRNAs. Line plots and violin plots were used to show fold changes and RPM expression levels, respectively (Fig. 3A to C and Fig. S2). We found that the trend of the three significant clusters was generally rising, whereas that of the five nonsignificant clusters was descending (Fig. S2A) or nondirectional (Fig. S2B). We named the three significantly upregulated clusters UP1, UP2, and UP3, which had 328, 237, and 524 circRNAs, respectively. Representative circRNAs of each cluster are listed in the bottom panels of Fig. 3A to C.

10.1128/mBio.03075-21.3 FIG S2 Time series trend analysis of differentially expressed cellular circRNAs upon RSV infection. Five nonsignificant temporal expression clusters (*P* > 0.05) were obtained with the 2,280 differentially expressed circRNAs by STEM analysis. (Left) Line plots were used to show fold changes (the number of circRNAs assigned to each cluster and the *P* values are shown). The gray lines indicate the profiles of individual circRNAs, and the black lines show the model profile for each cluster in the line plots. (Right) Violin plots were used to show the absolute expression levels (log_2_ scale). (A) Three clusters were generally descending. (B) Two clusters were generally nondirectional. Download FIG S2, PDF file, 2.5 MB. Copyright © 2021 Yao et al. 2021 Yao et al. https://creativecommons.org/licenses/by/4.0/ This content is distributed under the terms of the Creative Commons Attribution 4.0 International license.

We next performed gene ontology (GO) and KEGG pathway enrichment analyses for parental protein-coding genes using DAVID (25). In the analysis, we focused on the three significant clusters with a total of 1,089 cellular circRNAs from 871 parental source genes. The top 41 regulated GO processes (*P* < 0.01) (Fig. 3D) and the top 21 regulated KEGG pathways (*P* < 0.05) (Fig. 3E) were demonstrated and ranked according to their *P* values. The 871 genes demonstrated significant enrichment of GO terms related to the type I interferon (IFN) signaling pathway, the interferon gamma-mediated signaling pathway, positive regulation of the RIG-I signaling pathway, the defense response to virus, the innate immune response, positive regulation of NF-κB transcription factor (TF) activity, and viral process, etc. (Fig. 3D). They also demonstrated significant enrichment of KEGG pathways related to herpes simplex virus infection, Epstein-Barr virus infection, influenza A virus infection, the NF-κB signaling pathway, and hepatitis C virus infection, etc. (Fig. 3E). In summary, GO and KEGG pathway enrichment analyses indicate that circRNAs may be related to the innate immune response, the interferon signaling pathway, and pathways related to virus infection.

### Validation of the top 10 cellular circRNAs from the UP1 cluster and verification of their upregulation induced by RSV infection.

**FIG 4:**
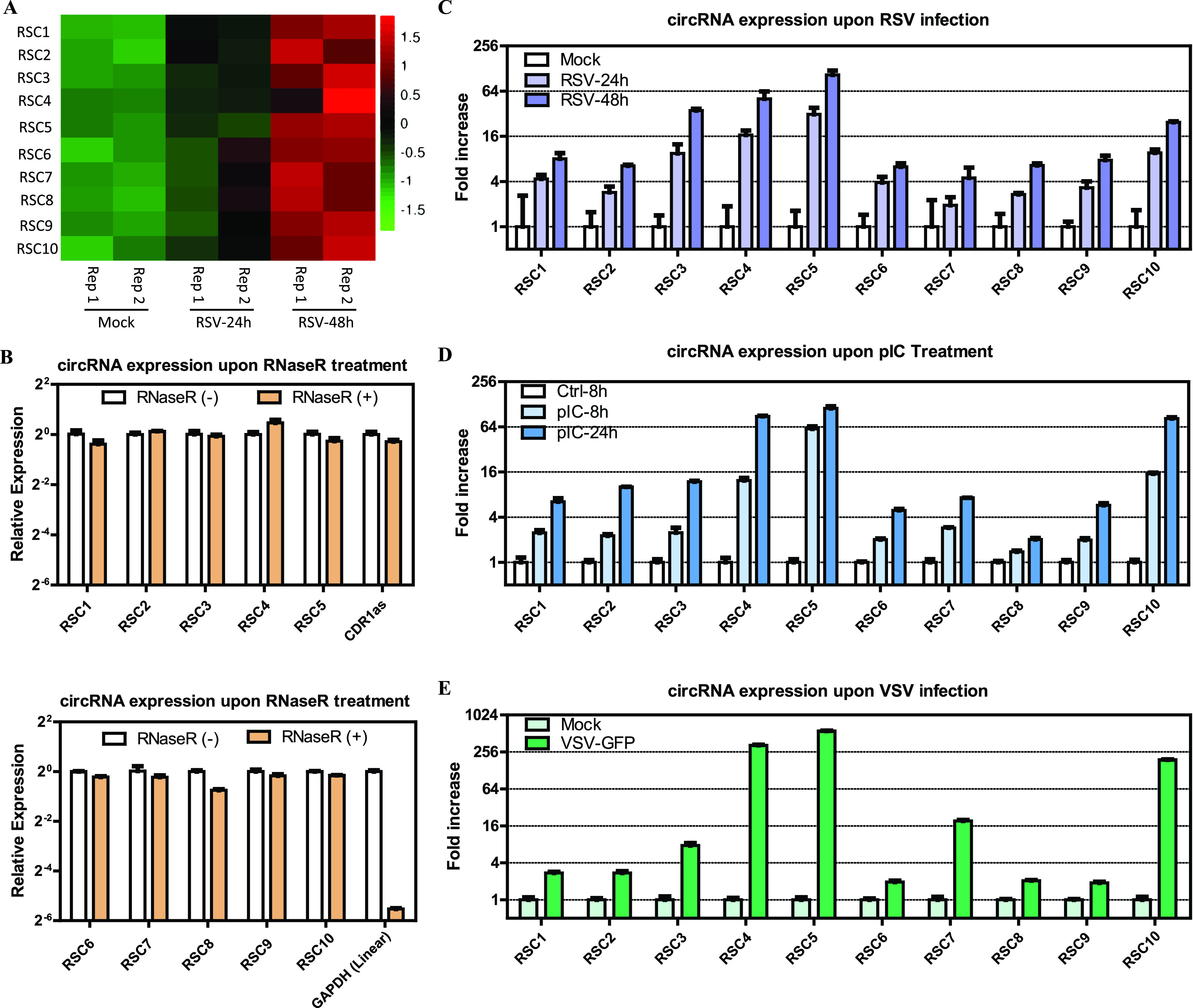
Validation of the top 10 RSCs from the UP1 cluster and effects of RSV infection, pIC, and VSV infection on the expression of the top 10 RSCs. (A) Heat map for the top 10 RSCs based on normalized RPM of RNA-seq data (log_10_ scale). (B) qRT-PCR analysis of the abundances of cellular circRNAs treated with RNase R. The amounts were normalized to the value measured in the untreated group. (C) Effect of RSV infection (MOI = 1) on the expression of the top 10 RSCs by qRT-PCR analysis. The amounts were normalized to the value measured in the mock infection group. (D) Effect of pIC on the expression of the top 10 RSCs. (E) Effect of VSV-GFP (green fluorescent protein) infection (MOI = 1) on the expression of the top 10 RSCs. Data in panels B to E are presented as means ± standard errors of the means (SEMs) (*n *= 3).

For further analysis, we focused on the UP1 cluster circRNAs that exhibited a steady increase upon RSV infection. We selected the top 10 circRNAs and called them RSV-stimulated circRNAs (RSCs). Cutoff filtering for selection was as follows: a >3-fold alteration of circRNA expression was demonstrated when the RSV 24-hpi group was compared with the mock group, and a >6-fold alteration of circRNA expression was demonstrated when the RSV 48-hpi group was compared with the RSV 24-hpi group. Detailed characteristics and a heat map of the expression of the 10 RSCs are shown in Table 2 and Fig. 4A, respectively. Divergent primers (Table S1A) were designed against the 10 circRNAs. Each primer pair amplified a single, distinct product of the expected size from RSV-infected A549 cells (Fig. S3A), and back-splicing junctions were further validated by Sanger sequencing (Fig. S3B). The enrichment of the 10 back-spliced circRNAs was apparent following RNase R treatment, whereas the abundance of linear glyceraldehyde-3-phosphate dehydrogenase (GAPDH) RNAs decreased (Fig. 4B).

**TABLE 2:** List of circRNAs validated by divergent primers

circRNA	circBase ID	Positions^*a*^	Strand	Genomic length (bp)	Spliced length (bp)	Gene symbol
RSC1	hsa_circ_0025721	chr12: 27368262–27390239	+	21,978	888	*ARNTL2*
RSC2	hsa_circ_0001964	chr12: 27368262–27380404	+	12,143	453	*ARNTL2*
RSC3	hsa_circ_0004662	chr6: 159682474–159688242	−	5,769	462	*SOD2*
RSC4	hsa_circ_0082624	chr7: 139061036–139074030	−	12,995	399	*ZC3HAV1*
RSC5	hsa_circ_0001426	chr4: 88475841–88494331	+	18,491	1,052	*HERC5*
RSC6	hsa_circ_0003922	chr2: 230442937–230450255	+	7,319	713	*SP100*
RSC7	hsa_circ_0134778	chr7: 8003908–8071131	+	67,224	720	*GLCCI1*
RSC8	hsa_circ_0008706	chr9: 110136127–110138539	+	2,413	2,413	*PALM2-AKAP2*
RSC9	hsa_circ_0007242	chr10: 13127745–13136897	+	9,153	523	*OPTN*
RSC10	hsa_circ_0082633	chr7: 139083780–139089759	−	5,980	389	*ZC3HAV1*
rsv_circ_449		RSV: 2348–2600	+	252	252	RSV P gene
rsv_circ_482		RSV: 2356–2597	+	241	241	RSV P gene
rsv_circ_664		RSV: 2879–3077	+	198	198	RSV P gene
rsv_circ_969		RSV: 5938–6921	+	983	983	RSV F gene
rsv_circ_305		RSV: 1571–1802	+	231	231	RSV N gene
rsv_circ_443		RSV: 2348–2580	−	232	232	RSV P gene
rsv_circ_526		RSV: 2368–2597	+	229	229	RSV P gene
rsv_circ_561		RSV: 2379–2605	+	226	226	RSV P gene

a chr, chromosome.

10.1128/mBio.03075-21.4 FIG S3 Validation of the top 10 cellular circRNAs from UP1 clusters using RT-PCR and Sanger sequencing. (A) RT-PCR products with divergent primers showing a single, distinct product of the expected size. (B) Sanger sequencing showing the back-spliced events of candidate circRNAs. Download FIG S3, PDF file, 1.1 MB. Copyright © 2021 Yao et al. 2021 Yao et al. https://creativecommons.org/licenses/by/4.0/ This content is distributed under the terms of the Creative Commons Attribution 4.0 International license.

10.1128/mBio.03075-21.10 TABLE S1 (A) Primer oligonucleotide and siRNA sequences used in this study. (B) TFBS prediction using AnimalTFDB3.0 and analysis of potential ORFs and IRESs using circRNADb and CSCD. For each RSC, the best transcripts (including the NCBI reference transcript identifier according to circBase and the converted Ensembl transcript identifier) and transcription factors (TFs) related to IFN that bind to RSC promoter/upstream regions predicted using AnimalTFDB3.0 are indicated. The potential ORFs predicted using both circRNADb and CSCD and the potential IRESs predicted using circRNADb are also indicated. Download Table S1, DOCX file, 0.04 MB. Copyright © 2021 Yao et al. 2021 Yao et al. https://creativecommons.org/licenses/by/4.0/ This content is distributed under the terms of the Creative Commons Attribution 4.0 International license.

We next validated the high expression levels of the 10 RSCs induced by RSV infection (MOI = 1) via quantitative real-time PCR (qRT-PCR), and the induced expression tendency was consistent with the RNA-seq results (Fig. 4C). Three of the RSCs (RSC3, RSC4, and RSC5) were induced more than 20-fold upon RSV infection, and RSC5 was induced more than 100-fold (Fig. 4C).

### Both poly(I·C) and another RNA virus also induced upregulation of the 10 RSCs from the UP1 cluster, and these RSCs generally inhibit RSV replication.

circRNAs may be upregulated by the antiviral response induced by RSV infection, and the above-described GO and KEGG pathway enrichment analyses, which showed that parental genes may be related to the innate immune response and the interferon signaling pathway, also supported this assumption. Given that double-stranded RNA (dsRNA) regions of viral genomes or viral replication can be detected by antiviral sensors and induce the expression of IFN and other antiviral genes, we investigated whether or not the dsRNA analogue poly(I·C) (pIC) could induce these RSCs’ upregulation. The results showed that all of the top 10 RSCs were significantly increased after transfection of A549 cells with pIC (Fig. 4D), and the fold changes were similar to those induced by RSV infection (Fig. 4C and D). As circRNAs were induced by pIC treatment, we speculated that they might also be induced after infection with other RNA viruses. Therefore, we evaluated circRNA levels in A549 cells infected with vesicular stomatitis virus (VSV), an RNA virus that replicates in the cytoplasm, which is similar to RSV, at an MOI of 1. As shown in Fig. 4E, VSV also induced the upregulation of 10 RSCs detected 24 h after infection.

**FIG 5:**
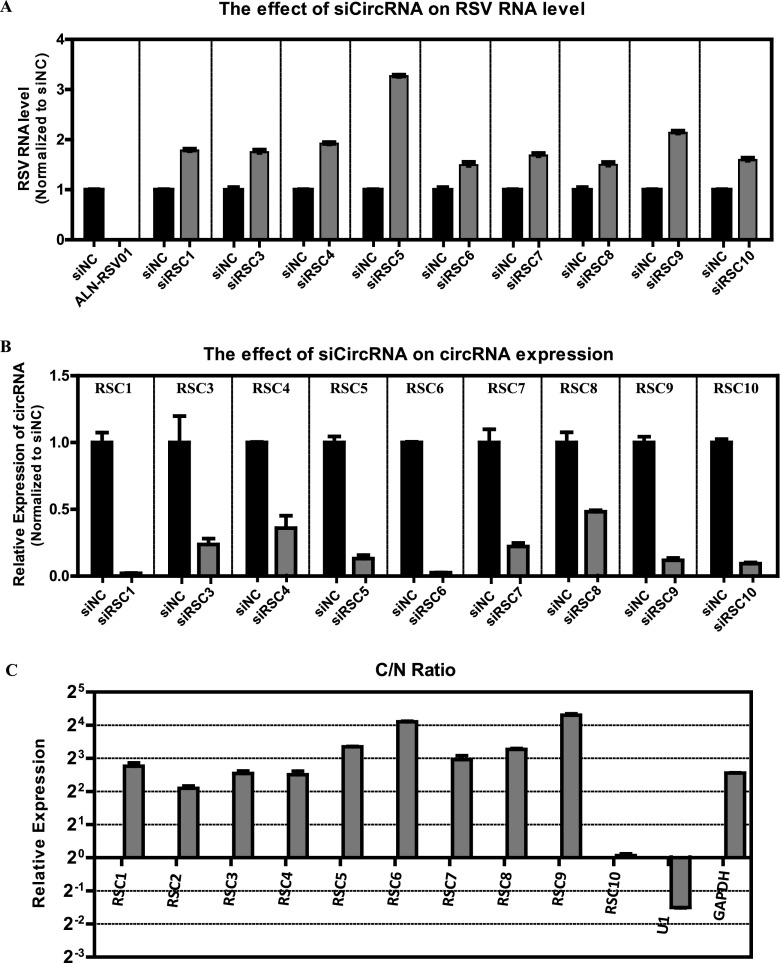
Analysis of the effects of the top 10 RSCs on RSV RNA levels in infected A549 cells and the subcellular localization of the 10 RSCs. (A) RSV RNA levels in infected A549 cells (MOI = 1) transfected with three pooled siRNAs for each circRNA (siCircRNA), with the exception of RSC2, assessed by qRT-PCR. (B) qRT-PCR analysis of the respective circRNAs in A549 cells treated with three pooled siRNAs as described above for panel A. In panels A and B, the RSV RNA levels or circRNA levels were normalized to the negative-control siRNA (siNC) group. (C) qRT-PCR data indicating the abundances of 10 RSCs in the subcellular localization (cytoplasm or nucleus) of A549 cells. The amounts of circRNAs were normalized to the value measured in the nucleus. C/N ratio, ratio of cytoplasm to nucleus.

We then investigated the effect of the top 10 RSCs on RSV infection in turn, and the subcellular localization of the 10 RSCs was also analyzed. The effect of the 10 RSCs on RSV infection was explored using RNA interference. We designed small interfering RNAs (siRNAs) for each circRNA (Table S1A), with the exception of RSC2, which we failed to design due to the special back-splice sequence. All the designed siRNAs targeted the back-splice sequence and therefore did not affect the expression of parental linear genes. A549 cells were transfected with pooled siRNAs prior to infection with RSV and collected at 48 hpi; the previously reported siRNA ALN-RSV01, which is directed against the RSV N protein and reduces RSV replication both *in vitro* and *in vivo* (26, 27), was transfected as a control. As expected, ALN-RSV01 efficiently impaired the RSV RNA level (Fig. 5A), and the pooled RSC-specific siRNAs efficiently decreased the levels of the respective circRNAs (Fig. 5B). Under these conditions, the RSV RNA levels were significantly increased by silencing all nine circRNAs, and RSC5 showed the most significant effect (Fig. 5A). Next, subcellular localization was evaluated via qRT-PCR in nuclear or cytoplasmic fractions of RSV-infected cells. As expected, U1 snRNA was enriched in the nuclear fraction, while the coding GAPDH mRNA was preferentially enriched in the cytoplasm; as demonstrated, nearly all of the top 10 circRNAs accumulated preferentially in the cytoplasm, and only RSC10 accumulated in both the cytoplasm and nucleus (Fig. 5C). In summary, the nine cellular circRNAs from the UP1 cluster generally inhibited RSV replication.

### Identification and profiling of viral circRNAs in RSV-infected A549 cells.

**FIG 6:**
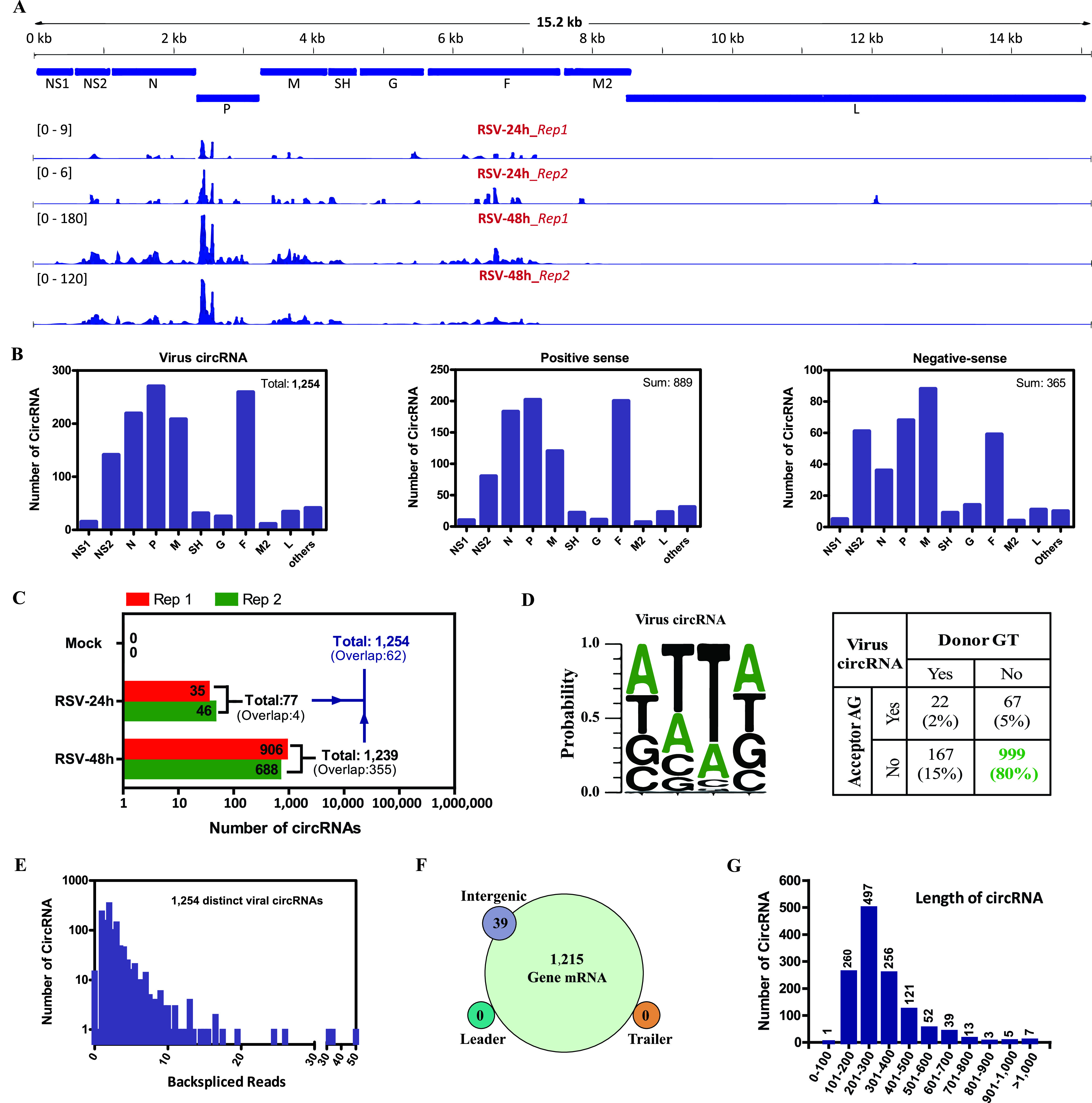
Profiling of viral circRNAs in RSV-infected A549 cells. (A) Integrative Genomics Viewer (IGV) displaying the coverage of candidate viral back-splice junctions aligning to the RSV genome identified in each RSV-infected sample. Standardized coverage depths (*y* axis) are indicated for each alignment. (B) Numbers of viral circRNAs mapped to each RSV gene, including general RSV RNA (left), positive-sense RSV RNA (middle), and negative-sense RSV RNA (right). (C) Length distribution of viral circRNAs. (D) Back-splice signals of viral circRNAs. (E) Numbers of viral circRNAs and back-spliced reads identified in all samples. (F) RSV genomic region annotations and numbers of viral circRNAs. (G) Length distribution of viral circRNAs.

Besides cellular circRNAs, we also investigated whether there are viral circRNAs derived from RSV. The pipeline used to identify cellular and viral circRNAs is illustrated in Fig. 1A. A total of 1,254 identified viral back-splice junctions of the RSV-infected samples were displayed using Integrative Genomics Viewer (IGV) (28) in reference to the RSV genome. The coverage depth is indicated on the *y* axis for each alignment panel (Fig. 6A). A total of 389 viral back-splice reads with high confidence, which were supported by more than 5 reads in RSV-infected samples, are further demonstrated in Fig. S4. As shown in Fig. 6A and Fig. S4, apparent coverage peaks or high coverage densities were observed across the P, N, NS2, M, and F genes of the RSV genome. We also analyzed the number of circRNAs for each RSV gene from the general RSV RNA (1,254 in total), positive-sense RNA/mRNA (889 in total), and negative-sense RNA (365 in total), and the results showed that the P, F, N, M, and NS2 genes were the top five genes with a high number of viral circRNAs (Fig. 6B). As shown in Fig. 6C, the number of viral circRNAs identified from each sample was demonstrated, with a total of 77 viral circRNAs from the RSV 24-h group and a total of 1,239 viral circRNAs from the RSV 48-h group.

10.1128/mBio.03075-21.5 FIG S4 Integrative Genome Viewer (IGV) display of viral back-splice junctions with high confidence identified. All the back-splice junctions were supported by more than 5 reads in RSV-infected A549 samples. Download FIG S4, PDF file, 1.1 MB. Copyright © 2021 Yao et al. 2021 Yao et al. https://creativecommons.org/licenses/by/4.0/ This content is distributed under the terms of the Creative Commons Attribution 4.0 International license.

Next, we profiled the characteristics of viral circRNAs (Fig. 6D to G). First, the splicing signals of viral circRNAs were investigated, and the results showed that most (80%) of the splicing signals did not have the canonical GT/AG donor/acceptor sequence, which is different from those of the cellular circRNAs, and the dominant splicing signals were replaced by the AT/TA donor/acceptor sequence (Fig. 6D). Second, the read distributions of viral circRNAs were analyzed, and 997 of the 1,254 viral circRNAs contained at least two unique back-spliced reads (Fig. 6E). Third, almost all of the viral circRNAs originated from RSV gene RNA, whereas only a small number of viral circRNAs aligned with intergenes, and no viral circRNAs mapped to the leader or trailer sequences (Fig. 6F). Finally, the average and median lengths of viral circRNAs were 316 and 262 nt, respectively, and the majority of viral circRNAs were less than 500 nt (Fig. 6G).

### Identification of temporally expressed clusters with 1,254 viral circRNAs and validation of the eight representative viral circRNAs.

**FIG 7:**
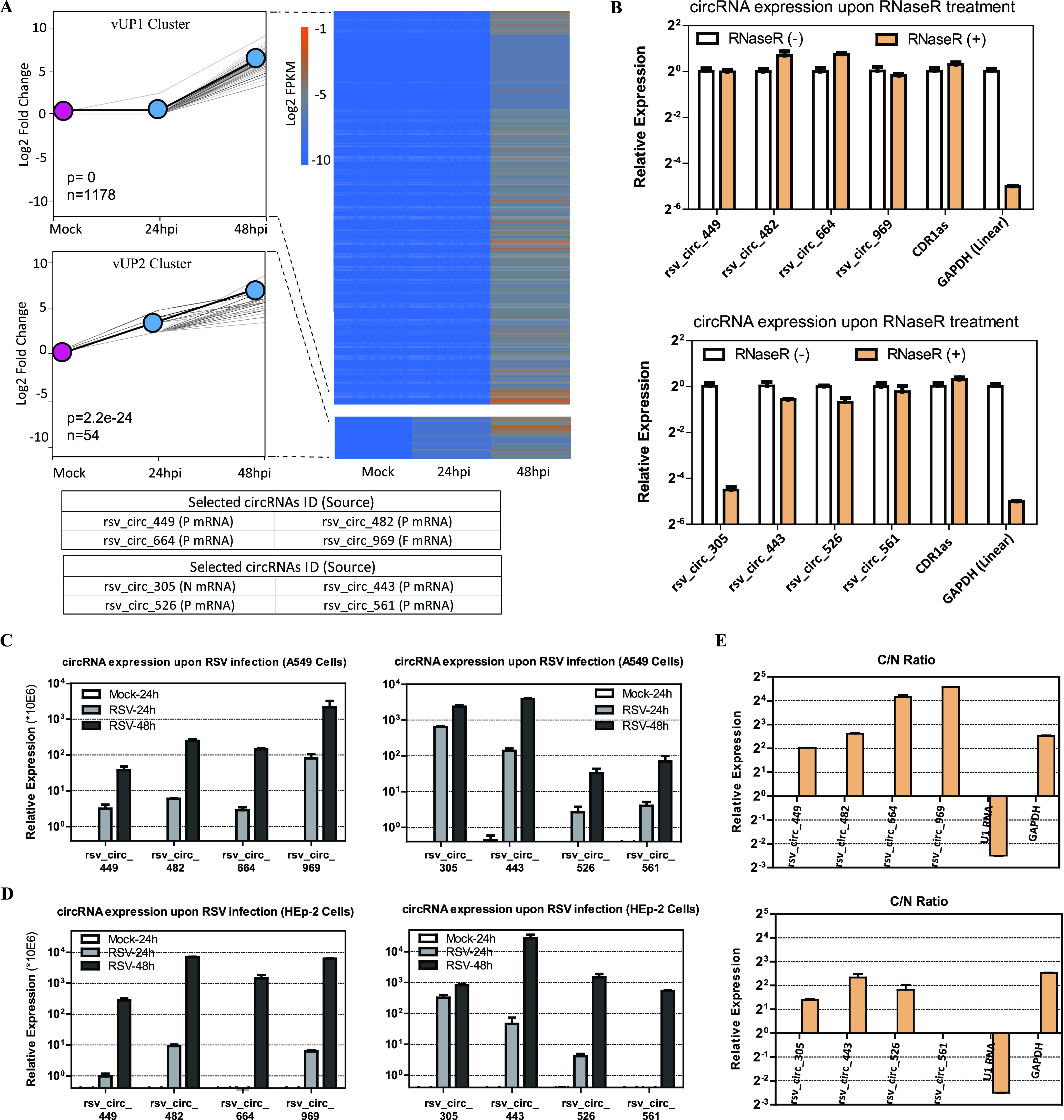
Trend analysis of viral circRNAs upon RSV infection and validation of the eight representative viral circRNAs from the vUP1 and vUP2 clusters. (A) Results showing two significant temporal clusters (*P* < 0.05) by STEM analysis. (Left) Line plots were used to show fold changes (the number of circRNAs assigned to each cluster and *P* values are shown). The gray lines indicate profiles of the individual circRNAs, and the black lines show the model profile for each cluster in the line plots. (Right) Heat map for viral circRNAs from the vUP1 and vUP2 clusters based on RPM of RNA-seq data (log_2_ scale). (Bottom) Representative circRNAs with the source of viral genes in parentheses for each cluster. (B) qRT-PCR analysis of the abundances of the eight viral circRNAs treated with RNase R. The amounts were normalized to the value measured in the untreated group. (C) Validation of the eight viral circRNAs’ upregulation upon RSV infection in A549 cells (MOI = 1) by qRT-PCR analysis. (D) Validation of the eight viral circRNAs’ upregulation upon RSV infection in HEp-2 cells (MOI = 1) by qRT-PCR analysis. In panels C and D, the amounts were normalized to the value measured in the mock infection group. (E) qRT-PCR data indicating the abundances of the eight viral circRNAs in the subcellular localization (cytoplasm or nucleus) of A549 cells. C/N ratio, ratio of cytoplasm to nucleus. In panels B to E, data are presented as means ± SEMs (*n *= 3).

Next, the expression of viral circRNAs in RSV-infected A549 cells was calculated, which was referenced to the cellular circRNAs’ expression and also based on RPM. We also performed STEM clustering with 1,254 viral circRNAs to demonstrate the dynamic expression of RSV-derived viral circRNAs. As expected, we obtained four generally rising temporal expression clusters because the expression of viral circRNAs depended on RSV RNA replication and viral production. Two of the four clusters were significant (Fig. 7A); we named the two significant clusters of viral circRNAs the vUP1 and vUP2 clusters, which had 1,178 and 54 circRNAs, respectively. For the vUP1 and vUP2 clusters, line plots and heat maps were used to demonstrate fold changes and log_2_ expression ratios of RPM, respectively; four representative viral circRNAs from each cluster are listed in the bottom panels, that is, rsv_circ_449/482/664/969 from the vUP1 cluster and rsv_circ_305/443/526/561 from the vUP2 cluster (Fig. 7A).

We next analyzed the eight viral circRNAs from the vUP1 and vUP2 clusters. Detailed characteristics of the eight viral circRNAs are shown in Table 2. Divergent primers were designed against the eight viral circRNA candidates, each primer pair amplified a single and distinct product of the expected size from RSV-infected A549 cells (Fig. S5A), and the back-splicing junctions of the eight viral circRNAs were further validated by Sanger sequencing (Fig. S5B). The enrichment of seven out of eight back-spliced events was apparent following RNase R treatment (Fig. 7B); however, the expression of rsv_circ_305 was significantly decreased upon RNase R treatment (Fig. 7B). Quantitative PCR (qPCR) validation has shown that some experimentally validated circRNAs are sensitive to RNase R (23, 29–31); it is unclear whether there are specific features or structures of these circRNAs that systematically result in RNase R sensitivity (32).

10.1128/mBio.03075-21.6 FIG S5 Validation of eight representative viral circRNA candidates using RT-PCR and Sanger sequencing. (A) RT-PCR products with divergent primers showing a single, distinct product of the expected size from cDNA of RSV-infected A549 cells. (B) Sanger sequencing showing the back-spliced events of candidate circRNAs. Download FIG S5, PDF file, 1.9 MB. Copyright © 2021 Yao et al. 2021 Yao et al. https://creativecommons.org/licenses/by/4.0/ This content is distributed under the terms of the Creative Commons Attribution 4.0 International license.

We then validated the expression trend of viral circRNAs upon RSV infection in A549 cells (MOI = 1) by qRT-PCR (Fig. 7C). Furthermore, we investigated the expression trends of the eight viral circRNAs upon RSV infection in HEp-2 cells (MOI = 1) (Fig. 7D). The qRT-PCR results showed that all eight viral circRNAs were significantly increased in RSV-infected A549 and HEp-2 cells (Fig. 7C and D). Subcellular localization analysis of the eight viral circRNAs was also performed, and the results showed that seven of the eight viral circRNAs accumulated in the cytoplasm, while only rsv_circ_561 accumulated in both the cytoplasm and nucleus (Fig. 7E).

Given that some viral circRNAs are derived from the RSV negative-sense genome and that some viral circRNAs are derived from the positive-sense antigenome or positive-sense mRNA, circRNA-specific reverse transcription primers were designed against rsv_circ_969 and rsv_circ_443, respectively (Table S1A) to verify that rsv_circ_969 is from positive-sense RNA/mRNA and that rsv_circ_443 is from negative-sense RNA. As illustrated in Fig. S6, rsv_circ_969 is predicted to be derived from positive-sense RSV F gene RNA/mRNA, which has an AA/AT donor/acceptor sequence, and rsv_circ_443 is predicted to be derived from negative-sense RSV P gene RNA, which has an AT/TA donor/acceptor sequence. The RT-PCR results with rsv_circ_969- or rsv_circ_443-specific reverse transcription primers showed that a single and distinct product of the expected size from RSV-infected A549 cells was amplified with each primer pair compared with mock-infected A549 cells, and the expected products were further validated by Sanger sequencing (data not shown).

10.1128/mBio.03075-21.7 FIG S6 Graphical presentation of the formation of rsv_circ_969 and rsv_circ_443. rsv_circ_969 is from positive-sense RNA or mRNA, and rsv_circ_443 is from negative-sense RNA. Download FIG S6, PDF file, 1.9 MB. Copyright © 2021 Yao et al. 2021 Yao et al. https://creativecommons.org/licenses/by/4.0/ This content is distributed under the terms of the Creative Commons Attribution 4.0 International license.

## DISCUSSION

This study is the first to characterize the cellular and viral circRNAomes induced by RSV infection in A549 cells, which is a widely used *in vitro* model of RSV infection of airway epithelial cells. First, from the perspective of the cellular circRNAome, we identified a large number of cellular circRNAs in RSV- and mock-infected A549 cells from diverse genomic locations. Filtering analysis identified 2,280 differentially expressed cellular circRNAs, and further STEM trend clustering with these differentially expressed circRNAs identified three significant expression clusters (UP1, UP2, and UP3) that exhibited generally rising trends upon RSV infection. Subsequent GO process and KEGG pathway enrichment analyses with parental source genes of 1,089 circRNAs from the three significant clusters revealed the most significant regulated GO processes and KEGG pathways. The actual existence of the top 10 RSCs from the focused UP1 cluster was validated by RT-PCR, Sanger sequencing, and RNase R resistance. Next, the increased expression of the 10 RSCs by RSV infection in A549 cells was validated by qRT-PCR; further results showed that pIC and VSV also induced the upregulation of the 10 RSCs. The siRNA results indicated that the concerned RSCs from the UP1 cluster generally inhibited RSV replication. Second, from the perspective of the viral circRNAome, we identified 1,254 viral circRNAs in RSV-infected A549 cells. As expected, STEM trend analysis with viral circRNAs demonstrated two significant clusters (vUP1 and vUP2). The actual existence of eight representative viral circRNAs from the vUP1 and vUP2 clusters was also validated with the same assays as those for cellular circRNAs, that is, single RT-PCR products, Sanger sequencing, and RNase R resistance. The expression of the eight circRNAs induced by RSV infection was validated by qRT-PCR in A549 cells and HEp-2 cells. Additionally, we confirmed that rsv_circ_969 in the vUP1 cluster is positive sense and that rsv_circ_443 in the vUP2 cluster is negative sense.

circRNAs are found across every domain of life, and they have been identified and characterized from archaea (33), plants (34), and animals (6). For viruses, DNA viruses, including EBV, KSHV, and HPV, were reported to express viral circRNAs recently (16–19). The present study further expands our contention that circular RNAs could be derived from an RNA virus, RSV. We identified 53,719 cellular circRNAs from the human genome (3.3 Gb long) and 1,254 viral circRNAs from the RSV RNA genome (15.2 kb long). It appears that the smaller RSV RNA genome produces more circRNAs per kilobase of genome length than the human genome. We further profiled the characteristics of cellular and viral circRNAs. First, from the perspective of genomic locations, our findings showed that the majority of cellular circRNAs were derived from exon circularization and that the majority of viral circRNAs were derived from RSV gene mRNA, both of which cover the open reading frame (ORF). Second, from the perspective of circRNA length, our results showed that the average and median lengths of cellular circRNAs (2,386 nt and 637 nt, respectively) (see Fig. S1C in the supplemental material) were longer than those of the viral circRNAs (316 nt and 262 nt, respectively) (Fig. 6G). Finally, the splicing signals were analyzed. Most reported circRNAs from eukaryotic cells are generated in a spliceosome-dependent manner (9), whereas the circularization process is independent of spliceosomes in archaea (33). Our results demonstrated that cellular circRNAs had a canonical GT/AG splicing donor/acceptor sequence (Fig. 2C), whereas viral circRNA had a flanking AT/TA donor/acceptor sequence but not a canonical GT/AG splicing sequence (Fig. 6D), which suggests that viral circRNAs of RSV formed by a mechanism that is independent of spliceosomes. RSV genome transcription and replication occur in the cytoplasm, and previous observations showed that RSV can grow even without nuclear involvement (1) and, however, that spliceosome-dependent splicing was in the nucleus; these observations also support the above-mentioned assumption that viral circRNAs of RSV formed not depending on spliceosomes.

We performed RNA pulldown assays coupled with mass spectrometry (MS) to elucidate the biogenesis mechanism of these viral circRNAs. It is known that RSV infection induces the formation of spherical cytoplasmic granules called inclusion bodies (IBs), which have been revealed to be viral factories where viral RNA synthesis (i.e., replication and transcription) occurs. RSV RNA synthesis is performed by the viral RNA-dependent RNA polymerase (RdRp), which is a complex of at least two proteins, the catalytic core L and the cofactor P. Several cellular proteins (such as heat shock protein 70/90 [HSP70/90] [35, 36] and actin [37]) proposed to be involved in the synthesis of RSV RNA were shown to colocalize within IBs (38). Interestingly, our MS results showed that the viral polymerase L was present in the viral circRNA-bound protein complex, and multiple HSP70s/HSP90s and actin were also present in the above-mentioned protein complex (data not shown). Based on these results, we hypothesized that viral proteins and assistant cellular proteins that participated in RSV RNA synthesis are also involved in the production of viral circRNAs. Inspired by this hypothesis, we further analyzed the flanking sequences of RSV mRNAs (Fig. S7A) (1), which were also synthesized by the viral RdRp. To our surprise, the dominant donor/acceptor-like flanking signals of RSV mRNAs were also the AT/TA sequences (Fig. S7A and B), and the probability summary of flanking signals between RSV mRNAs and viral circRNAs showed that they were strikingly similar (Fig. S7B and C). These results suggest that there may be specific flanking sequences that are employed by the viral RdRp for viral mRNA/circRNA generation. This is an interesting finding, and we will dig into it.

10.1128/mBio.03075-21.8 FIG S7 Donor/acceptor-like flanking sequences of RSV mRNAs and viral circRNAs. (A) Sequences of the gene start, gene end, and donor/acceptor-like flanking region of the 10 RSV genes. The main body of each gene is deleted and is represented by a box with the gene name. (B) Summary of flanking signals of RSV mRNAs. (C) Summary of flanking signals of viral circRNAs. Download FIG S7, PDF file, 0.5 MB. Copyright © 2021 Yao et al. 2021 Yao et al. https://creativecommons.org/licenses/by/4.0/ This content is distributed under the terms of the Creative Commons Attribution 4.0 International license.

In the current study, we found that RSV infection induced significant changes in cellular circRNA expression, and as demonstrated, the number of upregulated circRNAs was slightly higher than the number of downregulated circRNAs (Fig. 2D). By characterizing the dynamic changes in cellular circRNAs upon RSV infection, we identified 1,089 circRNAs with RSV-induced generally increasing expression patterns. Further results showed that both RSV and VSV infection induced the expression of the top 10 RSCs from the UP1 cluster, and pIC treatment exhibited similar inductions. Interestingly, the expressions of the 10 cellular circRNAs were all potentially regulated by IFN-related transcription factors (TFs) (interferon regulatory factor 3/7/9 [IRF3/7/9], STAT1/2, and NF-κB) (39), as the promoters of the best-matching mRNA transcripts of the 10 cellular circRNAs contain binding sites of IFN-related TFs predicted by AnimalTFDB3.0 (40) (Table S1B). These findings support the contention that these circRNAs are related to the antiviral response induced by RSV infection, as indicated by the GO and KEGG pathway enrichment analyses.

Subsequent investigation demonstrated that the top 10 RSCs from the UP1 cluster generally inhibited RSV replication. This result is reasonable because these circRNAs are assumed to be upregulated by the antiviral response induced by virus infection. Given that nearly all of the 10 circRNAs were abundant in the cytoplasm and that one of the most notable functions of cytoplasm circRNAs is the regulation of microRNA activity through sponging mechanisms, we mined online AGO2 photoactivatable ribonucleoside-enhanced crosslinking and immunoprecipitation (PAR-CLIP) or high-throughput sequencing of RNA isolated by crosslinking immunoprecipitation (HITS-CLIP) data from the doRiNA database (41) to determine if AGO2 occupies the region of these circRNAs. The mining results showed that 9 out of 10 circRNAs contain AGO2-binding sites and that 8 out of 10 circRNAs contain at least three AGO2-binding sites (Fig. S8). Therefore, these circRNAs possibly function by sponging microRNA. In addition, although circRNAs are believed to function mostly through noncoding mechanisms, some circRNAs have demonstrated protein-encoding capability but only when they contain ORF and internal ribosome entry site (IRES) elements (42). Xia et al. and Chen et al. developed online databases, the cancer-specific circRNA database (CSCD) (43) and circRNADb (44), respectively. The CSCD contains ORF information for 1,394,023 circRNAs from both tumor and normal samples, and circRNADb provides both IRES and ORF information for 32,914 human exonic circRNAs. We retrieved IRES and ORF information for the 10 RSCs from the two databases and found that RSC3, RSC5, RSC8, and RSC9 contain both IRESs and ORFs (Table S1B), indicating that these four circRNAs could generate translated products. Therefore, it cannot be excluded that these circRNAs function through coding proteins, but this hypothesis needs further investigation.

10.1128/mBio.03075-21.9 FIG S8 Predicted AGO2-binding sites from the doRiNA database were aligned to the circRNA sequences. (A) For RSC1 (hsa_circ_0025721): site 1 is chromosome 12 (chr12) positions 27521258 to 27521298, site 2 is chr12 positions 27523077 to 27523117, site 3 is chr12 positions 27543160 to 27543172, site 4 is chr12 positions 27538439 to 27538459, and site 5 is chr12 positions 27543037 to 27543058. (B) For RSC2 (hsa_circ_0001964), site 1 is chr12 positions 27521258 to 27521298, and site 2 is chr12 positions 27523077 to 27523117. (C) For RSC3 (hsa_circ_0004662), site 1 is chr6 positions 160105952 to 160106016, site 2 is chr6 positions 160103454 to 160103519, site 3 is chr6 positions 160109196 to 160109219, site 4 is chr6 positions 160105918 to 160105938, site 5 is chr6 positions 160105905 to 160105945, site 6 is chr6 positions 160103646 to 160103670, site 7 is chr6 positions 160103506 to 160103511, site 8 is chr6 positions 160109197 to 160109219, site 9 is chr6 positions 160105908 to 160105938, and site 10 is chr6 positions 160105908 to 160105938. (D) For RSC4 (hsa_circ_0082624), site 1 is chr7 positions 138758683 to 138758710, site 2 is chr7 positions 138758738 to 138758759, site 3 is chr7 positions 138758736 to 138758776, and site 4 is chr7 positions 138745782 to 138745835. (E) For RSC5 (hsa_circ_0001426), site 1 is chr4 positions 89407272 to 89407298, site 2 is chr4 positions 89408244 to 89408284, and site 3 is chr4 positions 89408258 to 89408285. (F) For RSC6 (hsa_circ_0003922), site 1 is chr2 positions 231307734 to 231307764, site 2 is chr2 positions 231308968 to 231308998, and site 3 is chr2 positions 231309029 to 231309054. (G) For RSC7 (hsa_circ_0134778), site 1 is chr7 positions 8043587 to 8043627, site 2 is chr7 positions 8043643 to 8043668, site 3 is chr7 positions 8095138 to 8095181, site 4 is chr7 positions 8099759 to 8099784, site 5 is chr7 positions 8099850 to 8099858, site 6 is chr7 positions 8110579 to 8110600, site 7 is chr7 positions 8043592 to 8043632, site 8 is chr7 positions 8095139 to 8095179, site 9 is chr7 positions 8099740 to 8099780, site 10 is chr7 positions 8110691 to 8110731, site 11 is chr7 positions 8095081 to 8095162, site 12 is chr7 positions 8099734 to 8099804, site 13 is chr7 positions 8099834 to 8099881, site 14 is chr7 positions 8043547 to 8043573, site 15 is chr7 positions 8043629 to 8043659, site 16 is chr7 positions 8043643 to 8043663, and site 17 is chr7 positions 8099838 to 8099858. (H) For RSC8 (hsa_circ_0008706), site 1 is chr9 positions 112898493 to 112898520, site 2 is chr9 positions 112898625 to 112898635, site 3 is chr9 positions 112900166 to 112900182, site 4 is chr9 positions 112900249 to 112900272, site 5 is chr9 positions 112898526 to 112898643, site 6 is chr9 positions 112900136 to 112900212, site 7 is chr9 positions 112898600 to 112898630, site 8 is chr9 positions 112899019 to 112899039, site 9 is chr9 positions 112899498 to 112899525, site 10 is chr9 positions 112899877 to 112899895, site 11 is chr9 positions 112899985 to 112900008, site 12 is chr9 positions 112900161 to 112900191, and site 13 is chr9 positions 112900436 to 112900463. (I) For RSC10 (hsa_circ_0082633), site 1 is chr7 positions 138768677 to 138768717. Download FIG S8, PDF file, 0.7 MB. Copyright © 2021 Yao et al. 2021 Yao et al. https://creativecommons.org/licenses/by/4.0/ This content is distributed under the terms of the Creative Commons Attribution 4.0 International license.

Although RSV was discovered more than 60 years ago, our understanding of the extent and diversity of the RSV transcriptome is limited. In this study, we report that RSV expresses a repertoire of a class of ncRNAs, namely, circRNAs. First, among the 1,254 viral circRNAs, 889 were from the positive-sense antigenome or mRNA, which is more than the 365 negative-sense circRNAs. This result is consistent with the fact that the antigenome RNA and mRNA of RSV are more abundant than genome RNA. Second, the RSV genome has 10 genes in the order 3′ NS1-NS2-N-P-M-SH-G-F-M2-L, in which the encoded proteins of the N, P, M, F, and L genes have clear orthologues throughout the *Paramyxoviridae*, and their relative genome order is conserved. Our results showed that the N, P, M, F, and NS2 genes were the top five genes with a large number of viral circRNAs. These observations suggest that N, P, M, and F are important for RSV infection, but although L is the largest gene and functionally conserved throughout the *Paramyxoviridae*, the number of viral circRNAs from the L gene was very small. Third, some of the RSV-derived circRNAs were expressed at levels that are comparable to or higher than those of the majority of cellular circRNAs. This finding supports the contention regarding the potential functional role of viral circRNAs. Finally, the eight representative viral circRNAs were upregulated by RSV infection in both A549 cells and HEp-2 cells, suggesting their roles in fundamental processes during viral infection. Together, these findings reveal a spectrum of RSV circRNAs with potential functional roles in RSV infection.

Overall, we revealed a new layer of host-virus interactions with circRNAs: RSV infection induced the differential expression of cellular circRNAs, some of which affected RSV infection, and RSV could also express viral circRNAs. These cellular or viral circRNAs may be novel biomarkers or therapeutic targets.

## MATERIALS AND METHODS

### Library construction and RNA sequencing.

After extraction, total RNAs were subjected to rRNA removal and then treated with RNase R to degrade the linear RNAs prior to library preparation. Next, a strand-specific library was constructed and sequenced using the Illumina HiSeq 2500 platform. Library construction and RNA sequencing were performed at the Gene Denovo Biotechnology Company (Guangzhou, China).

### Cell cultures and treatments.

A549 cells (catalogue no. TCHu150; The Chinese Academy of Sciences Cell Bank) and HEp-2 cells (ATCC CCL-23) were grown in Ham’s F-12K medium (catalogue no. 21127-022; Invitrogen) and Dulbecco’s minimal essential medium (DMEM), respectively, and both media were supplemented with 10% fetal bovine serum (FBS; HyClone) and 1% penicillin-streptomycin. The cells were maintained at 37°C in a humidified atmosphere containing 5% CO_2_.

pIC was purchased from Sigma (catalogue no. P9582). A549 cells were seeded into 6-well plates 16 h before treatment, and each 6-well plate then received 10 μg of pIC by transfection. Transfection was performed with 3.75 μl of Lipofectamine 3000 (Invitrogen) in 250 μl Opti-MEM medium (Gibco).

### Viruses, stock preparation, and viral infections.

RSV (A2 strain) was provided by Zifeng Yang (First Affiliated Hospital of Guangzhou Medical University, Guangzhou, China), and viruses were grown in HEp-2 cells for large-scale preparation. VSV was provided by Mingzhou Chen (State Key Laboratory of Virology and Modern Virology Research Center, Wuhan University), and viruses were grown in Vero cells for large-scale preparation. For viral infections, A549 or HEp-2 cells were washed with phosphate-buffered saline (PBS) and incubated with the viral inoculum in medium (2% FBS) for 90 min at 37°C at the indicated MOIs; after that time, the inoculum was removed, and fresh medium was added.

### RSV titration.

To determine the viral titer, HEp-2 cell monolayers were incubated with serial dilutions of the virus supernatants for 90 min at 37°C and then overlaid with medium containing 2% FBS and 1% methylcellulose. At 5 days postinfection (dpi), the cells were fixed with 10% (vol/vol) formaldehyde for 10 min, subsequently stained with 1% crystal violet for 20 min, and then washed with running water. Plaques were visualized and counted.

### Preparation of cell extracts, Western blotting, and antibodies.

Proteins were extracted from cells using radioimmunoprecipitation assay buffer supplemented with protease inhibitors (Sigma), with GAPDH as an internal control. For Western blotting, equal amounts of protein extracts boiled with a sodium dodecyl sulfate (SDS) loading buffer were subjected to SDS-polyacrylamide gel electrophoresis and then transferred onto a nitrocellulose membrane (Portran). The membrane was subsequently incubated with the respective primary antibodies. Horseradish peroxidase (HRP)-conjugated secondary antibodies were used to visualize the respective proteins with an enhanced chemiluminescence detection system (Millipore). The following primary antibody was used for Western blotting: monoclonal mouse anti-RSV fusion protein antibody (catalogue no. sc-101362; Santa Cruz).

### RNA preparations.

Total RNA was extracted from cells using TRIzol (Invitrogen) according to the manufacturer’s instructions. Nuclear and cytoplasmic RNAs were isolated using the Paris kit (catalogue no. AM1921; Invitrogen) according to the vendor’s protocol.

### Reverse transcription, PCR, and real-time PCR.

RNA was reverse transcribed by using the PrimeScript RT reagent kit with gDNA Eraser (catalogue no. RR047A; TaKaRa) in the presence of random hexamers (TaKaRa). The cDNA was used to perform PCR or qPCR using a TB green premix ExTaq II kit (catalogue no. RR820A; TaKaRa). The primers used are listed in Table S1A in the supplemental material. The housekeeping 18S rRNA or GAPDH gene served as an internal control. All reactions were run in triplicate using the Applied Biosystems 7500 real-time PCR system.

### RNase R resistance analysis.

Ten micrograms of total RNA was incubated for 20 min at 37°C with or without 2 U of RNase R (catalogue no. RNR07250; Epicentre) per μg of RNA, and therefore, the total amount of RNase R for 10 μg of RNA was 20 U. The resulting RNA was then purified with an RNeasy MinElute clean-up kit (catalogue no. 74204; Qiagen).

### Time series analysis to identify circRNAs across RSV infection.

circRNAs by the criteria of a fold change of >2.0 or <0.5 and a *P* value of <0.05 between the two compared groups of three (mock, 24 hpi, and 48 hpi) were taken for further analysis. In total, 2,208 circRNAs were obtained for STEM clustering analysis. STEM clustering, which is specifically designed to handle short time series gene expression data, was used to find the significant temporal patterns in RSV-associated circRNAs. Significant temporal expression patterns were obtained from expression profiles of RSV-associated circRNAs at two different time points (24 hpi and 48 hpi) by assuming the mock infection sample as a base time point. The clustered patterns with *P* values of <0.05 were considered significant patterns. There were 1,089 (out of 2,208) circRNAs showing significant temporal expression patterns and 1,119 (out of 2,208) circRNAs showing nonsignificant temporal expression patterns across RSV infection, as shown in Fig. 3A to C and Fig. S2, respectively.

### Bioinformatic analysis tools.

circBase (21) was used to annotate the identified cellular circRNAs in A549 cells. Viral circRNAs were displayed by IGV in reference to the RSV genome (GenBank nucleotide sequence accession no. M74568.1). The GO and KEGG pathway gene annotation enrichment analyses were performed using DAVID (https://david.ncifcrf.gov/) (25, 45). Venn diagrams were generated using an online analysis platform (http://bioinfogp.cnb.csic.es/tools/venny/index.html). The PCA, cluster, and trend analyses were performed using OmicShare tools (Gene Denovo).

10.1128/mBio.03075-21.1 TEXT S1 Detailed methods about AGO2-binding sites from CLIP data sets, transcriptional factor binding site (TFBS) prediction, and ORF and IRES predictions. Download Text S1, DOCX file, 0.04 MB. Copyright © 2021 Yao et al. 2021 Yao et al. https://creativecommons.org/licenses/by/4.0/ This content is distributed under the terms of the Creative Commons Attribution 4.0 International license.
