# Supplementary figures and images for: The Cellular and Viral circRNAome Induced by Respiratory Syncytial Virus Infection
Source: mBio. 2021 Dec 7;12(6):e03075-21. doi: 10.1128/mBio.03075-21 (PMC8649777; doi:10.1128/mBio.03075-21)

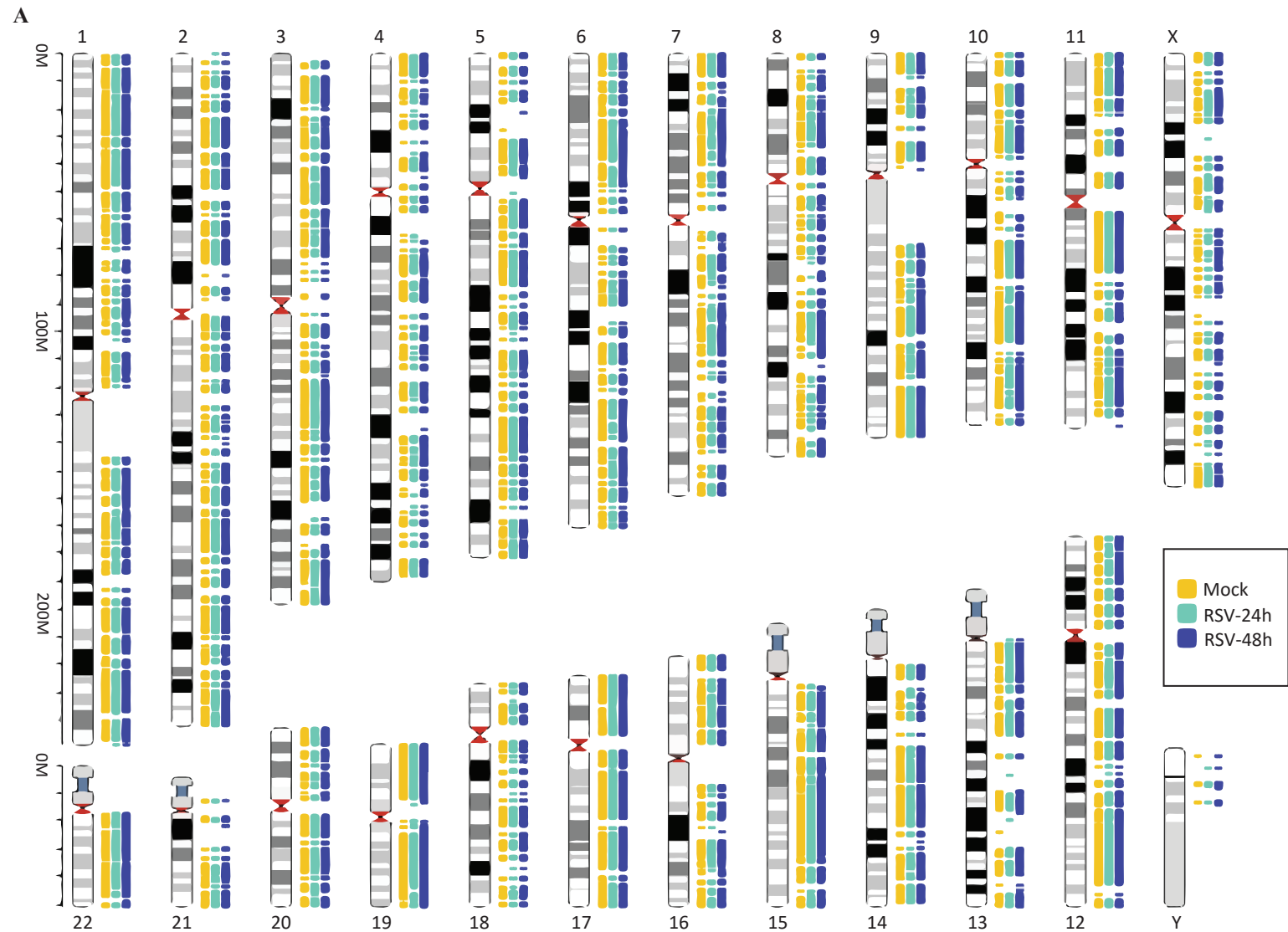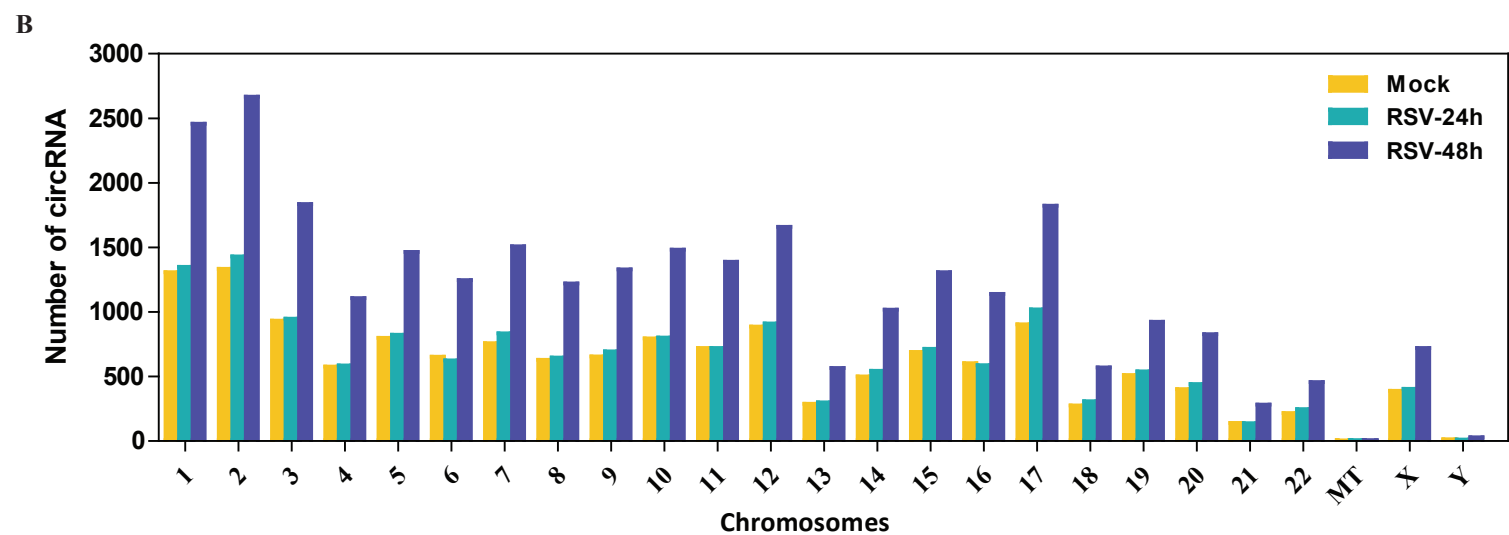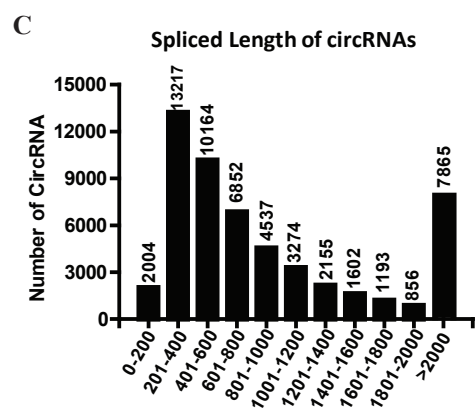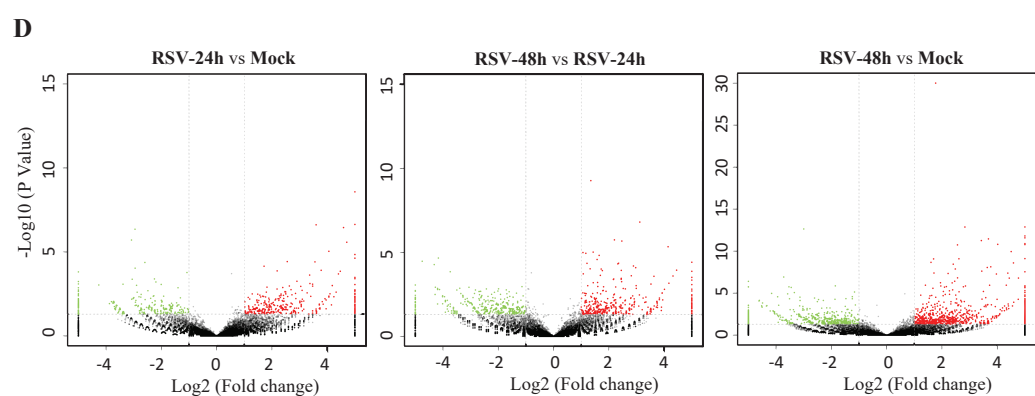

Supp Figure 1

Supplement: FIG S1 [file mbio.03075-21-sf001.pdf]

**A**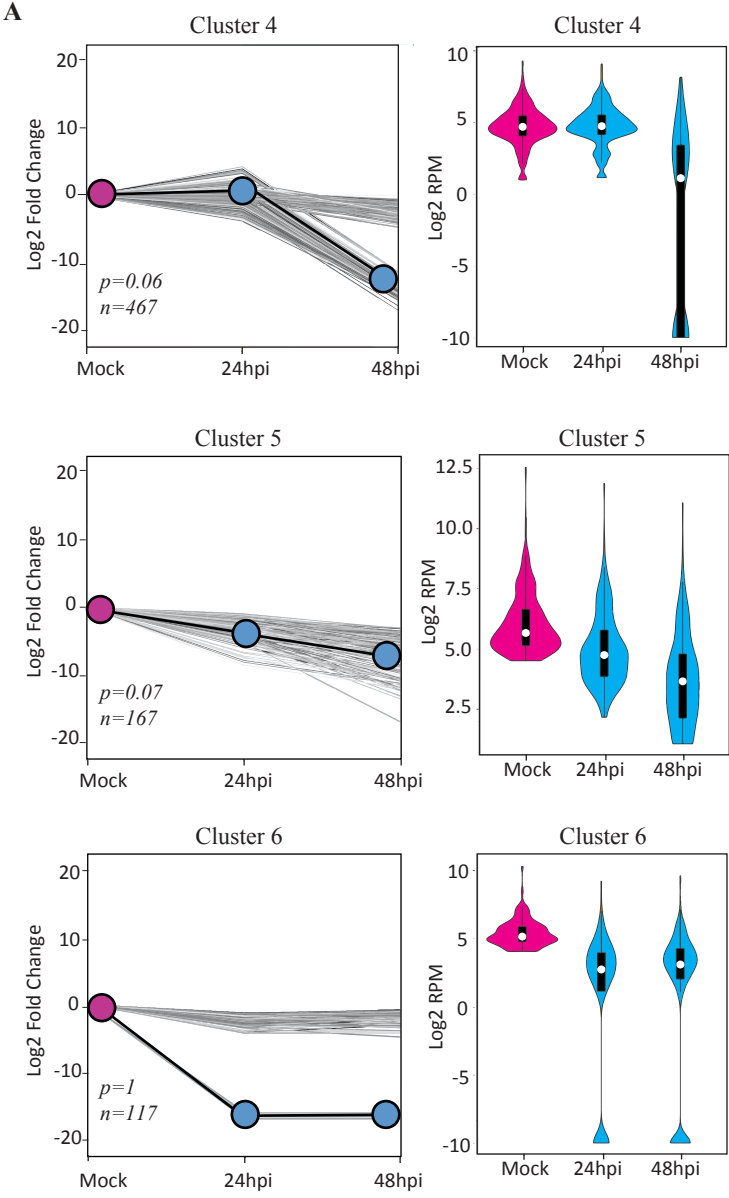**B**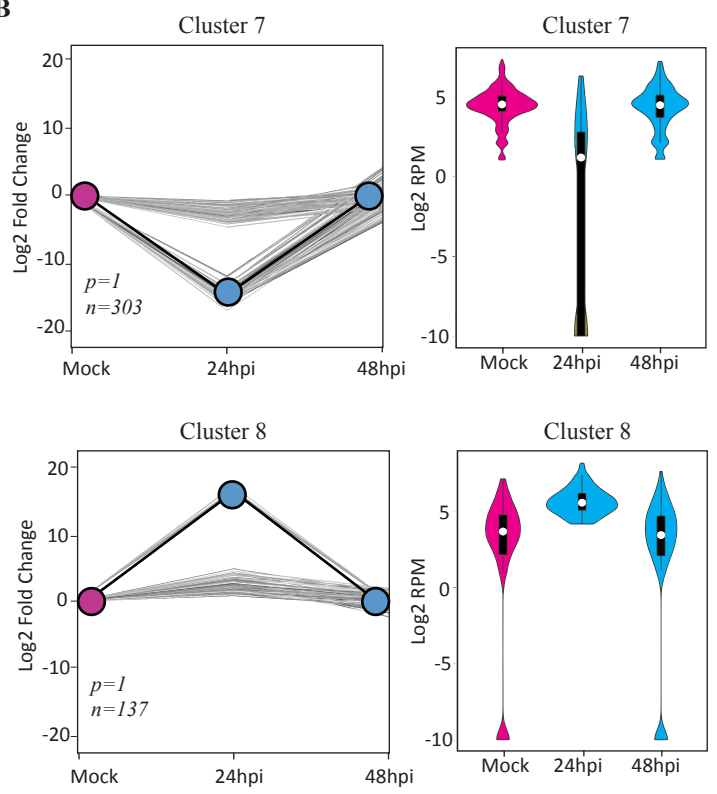**Supp Figure 2**

Supplement: FIG S2 [file mbio.03075-21-sf002.pdf]

**A**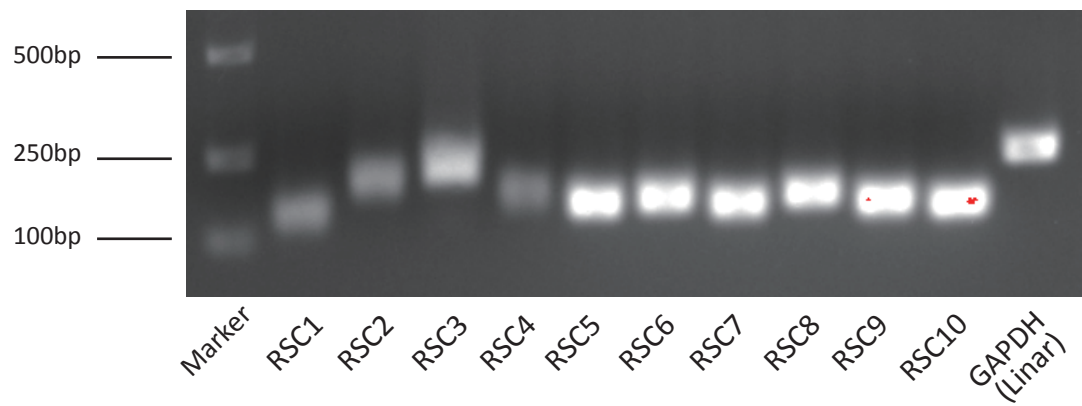**B**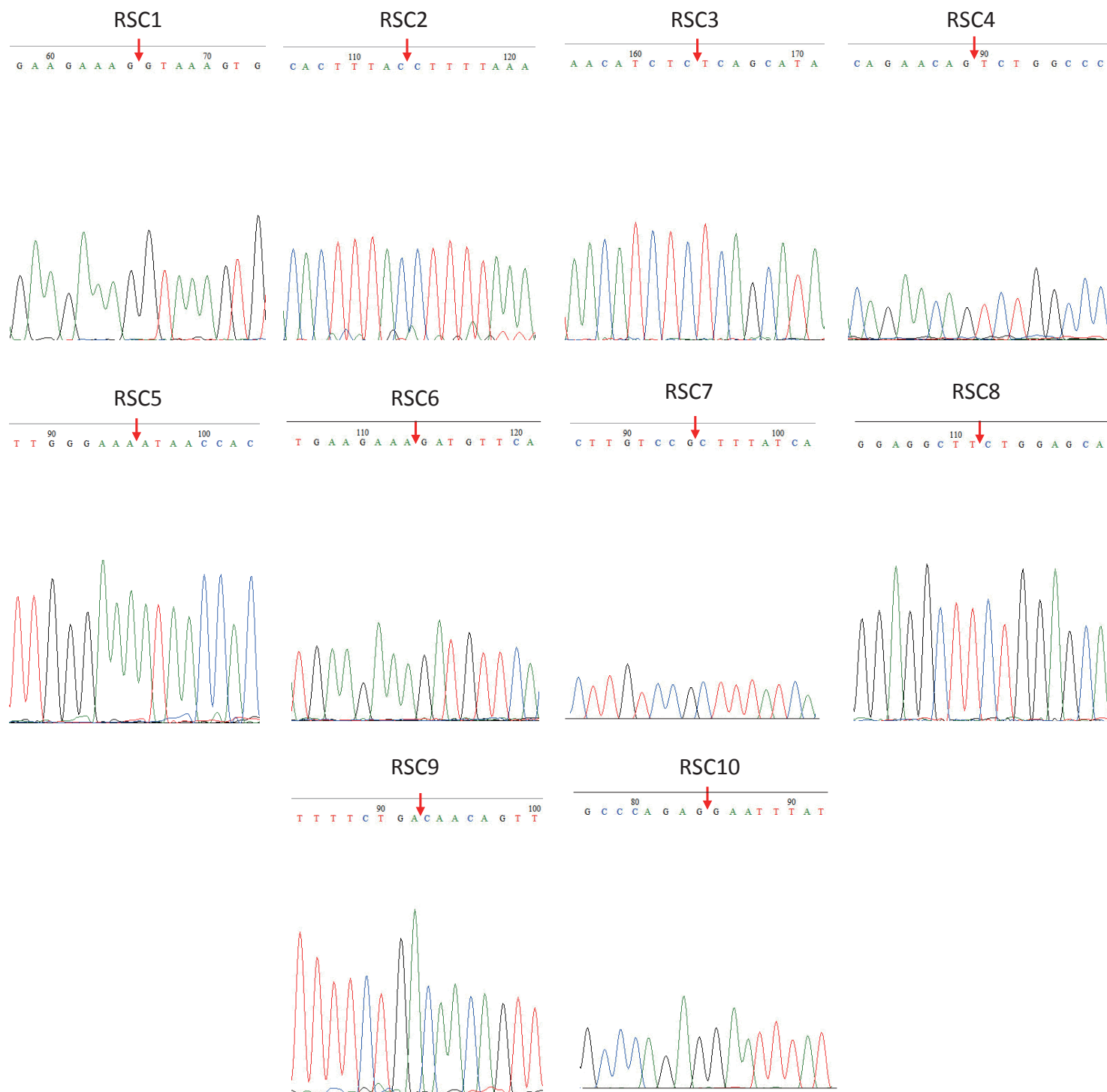**Supp Figure 3**

Supplement: FIG S3 [file mbio.03075-21-sf003.pdf]

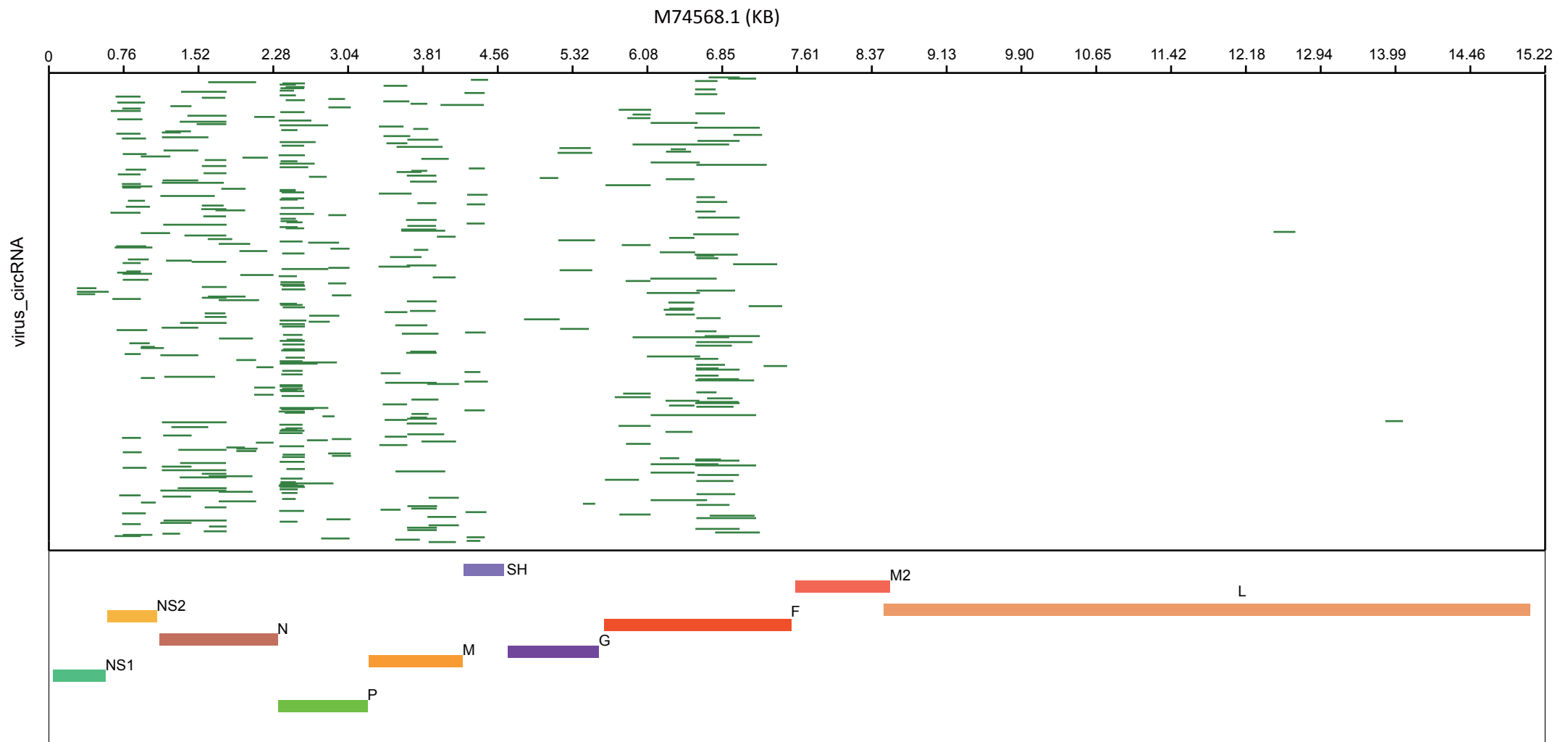

Supp Figure 4

Supplement: FIG S4 [file mbio.03075-21-sf004.pdf]

**A**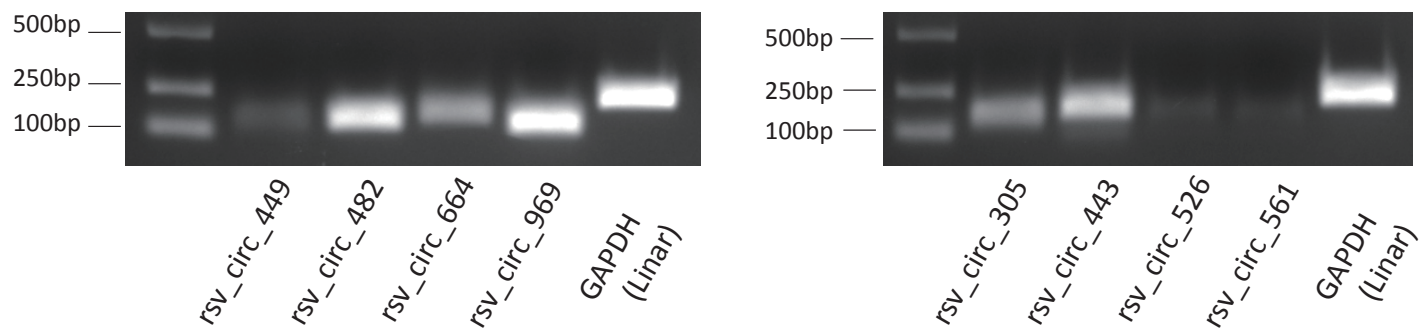**B**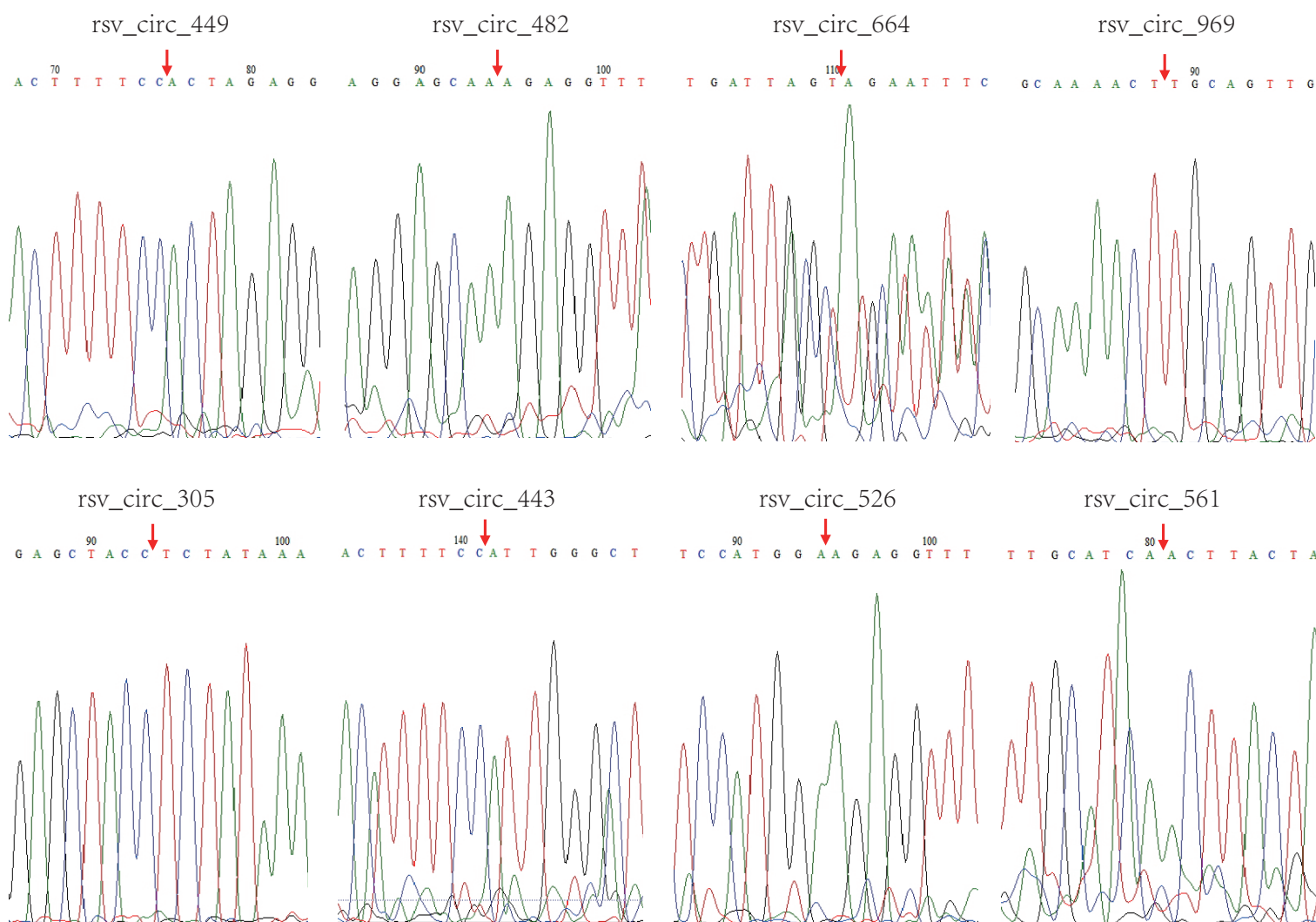**Supp Figure 5**

Supplement: FIG S5 [file mbio.03075-21-sf005.pdf]

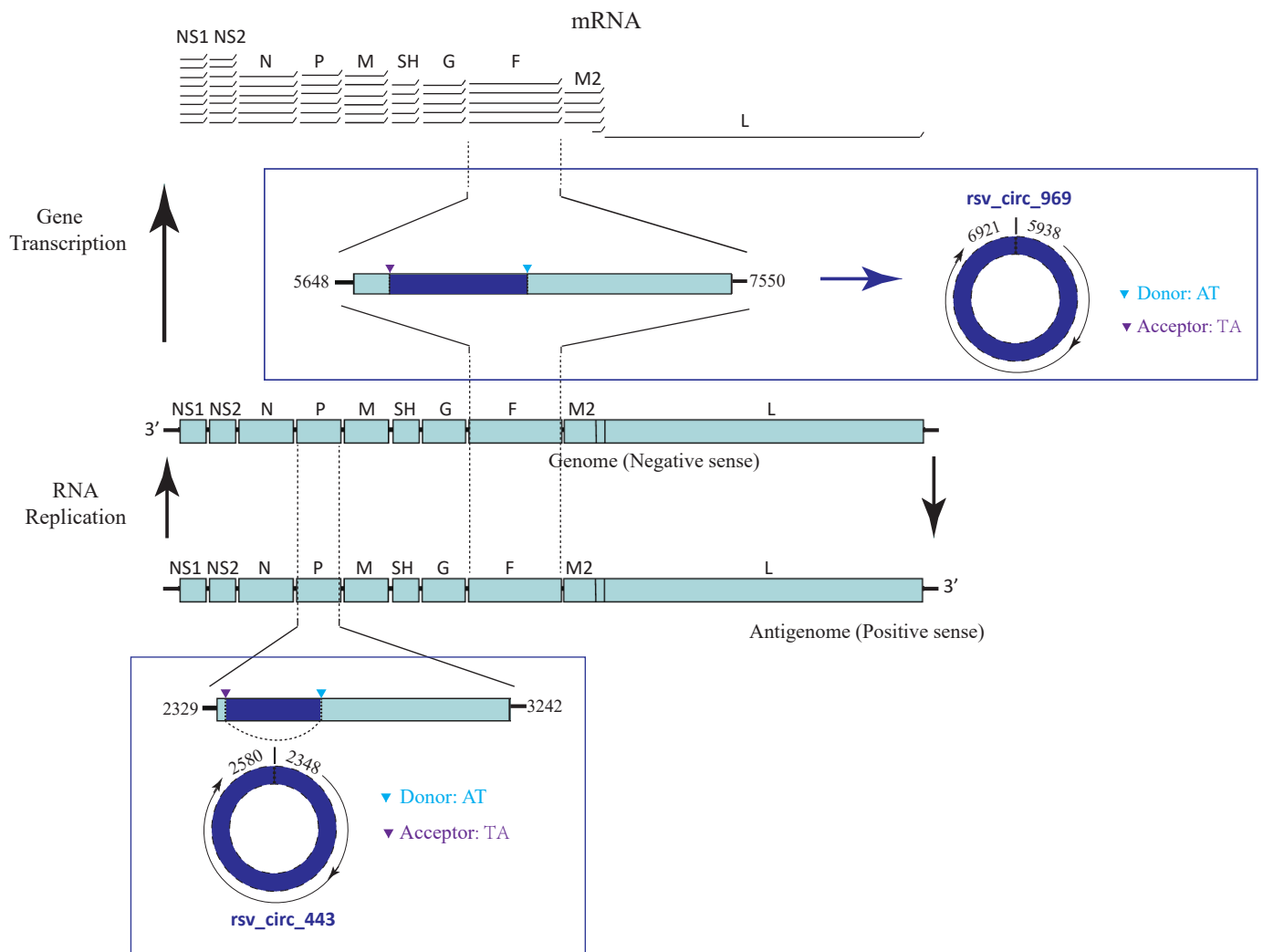

Supp Figure 6

Supplement: FIG S6 [file mbio.03075-21-sf006.pdf]

A

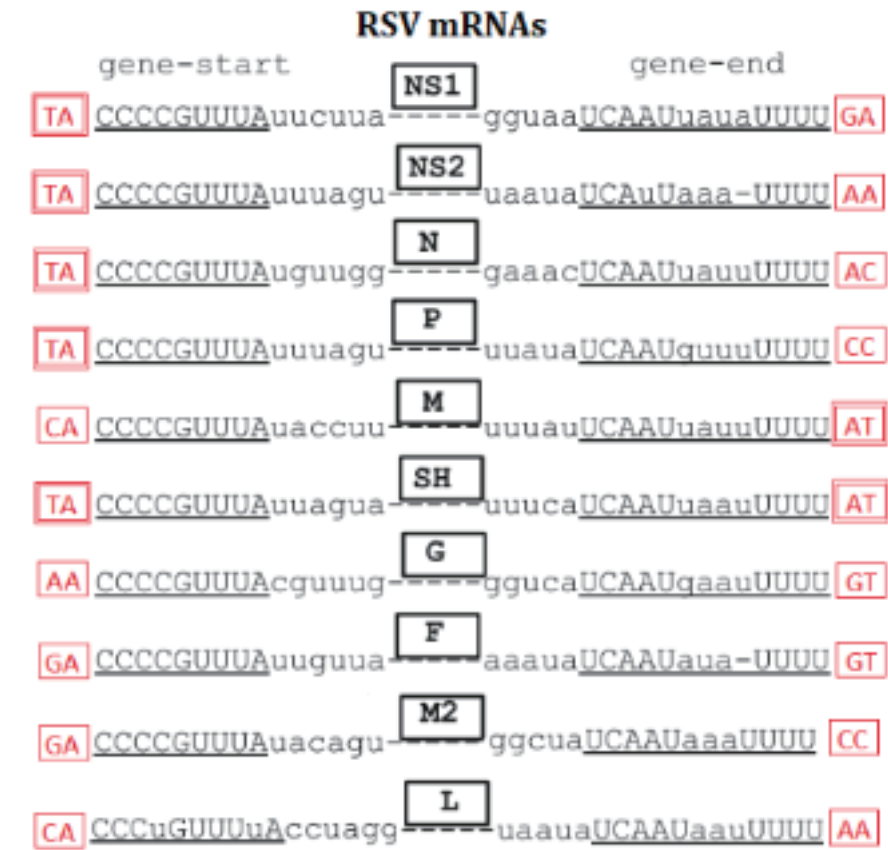

B

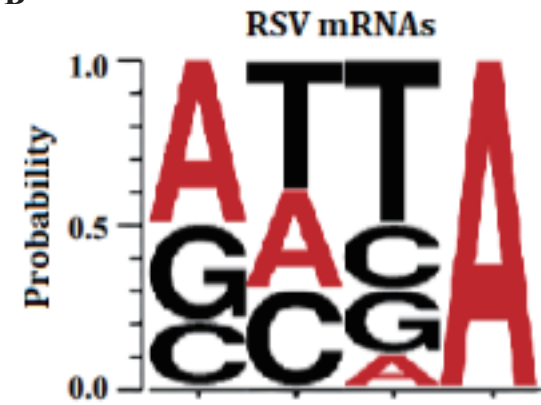

C

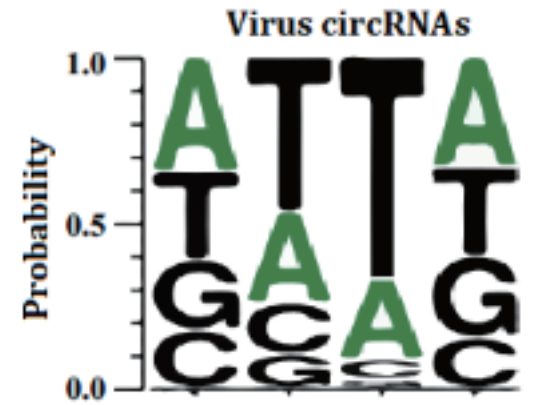

Supp Figure 7

Supplement: FIG S7 [file mbio.03075-21-sf007.pdf]

A

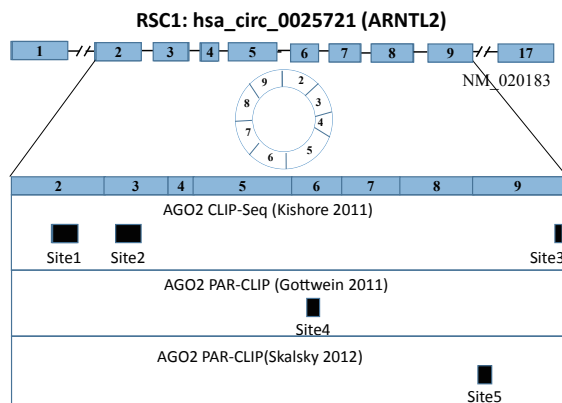

E

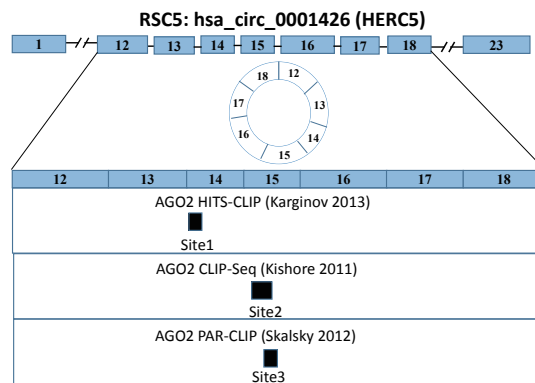

B

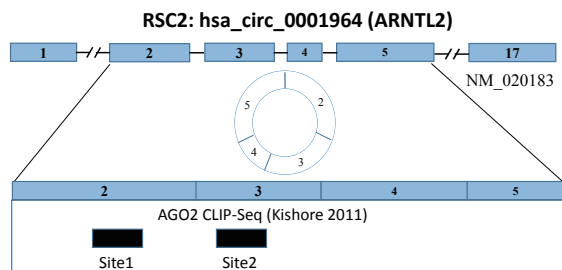

F

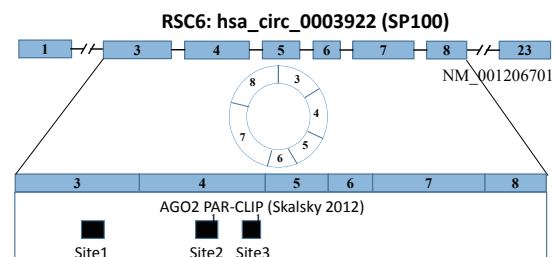

C

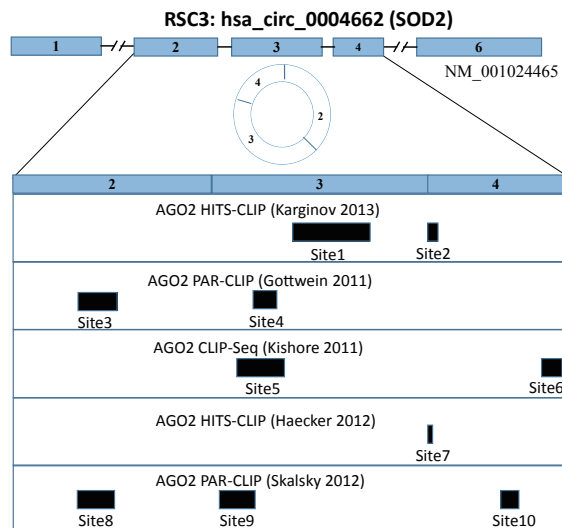

G

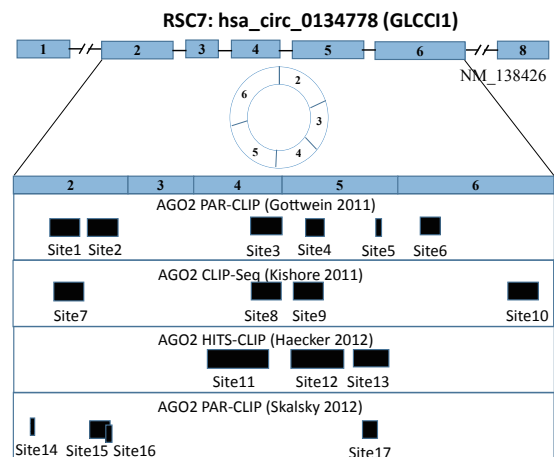

D

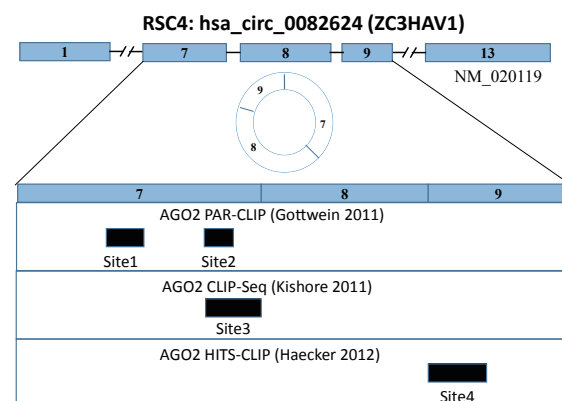

H

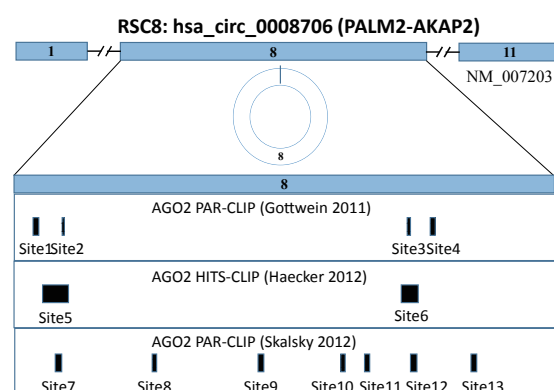

I

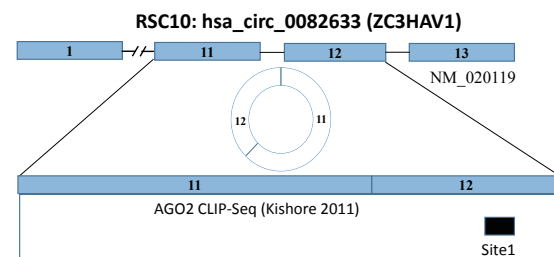

Supp Figure 8

Supplement: FIG S8 [file mbio.03075-21-sf008.pdf]
