# Supplementary material for: The Cellular and Viral circRNAome Induced by Respiratory Syncytial Virus Infection
Source: mBio. 2021 Dec 7;12(6):e03075-21. doi: 10.1128/mBio.03075-21 (PMC8649777; doi:10.1128/mBio.03075-21)
Supplement: TABLE S1 [file mbio.03075-21-st001.docx]

**Supplementary Table 1A** Primer and siRNA sequences used in this study

| **Alias Name** | **Sequences (5'--> 3')** |
| --- | --- |
| RSC1 | F: tattctggctcaagacgat; R: tctctcaacactttacctttc |
| RSC2 | F: aagagagggccagactgttc; R: tggtggtgaagacagaattggc |
| RSC3 | F: cagtatggagaagaggaatt; R: ttttgcatatctcgggcata |
| RSC4 | F: ctacaaaactgctccagaagc; R: cattctgcaacagacacctcaat |
| RSC5 | F: tgtgcacatcctcgctgtca; R: ggtccacatcatcctctgct |
| RSC6 | F: cacgatcgttatgctgagaga; R: agccgtcagcttctcctta |
| RSC7 | F: atgatcgtgataaagcggac; R: ggtagtgaacatgaggatc |
| RSC8 | F: acagaaaggtgctggccat; R: gagaatatttgcataaattcctctg |
| RSC9 | F: cctccccaaaactgttgtcag; R: cctgcttggcaatggtttgct |
| RSC10 | F: agcagcagacagattccac; R: gtggtcctttcacatccagt |
| rsv_circ_449 | F: gaaaacctctagtggaaaag; R: tcttgggatctttgggtgat |
| rsv_circ_482 | F: caaagaaaacctctttgctcc; R: gagttgacagatatgatactatc |
| rsv_circ_664 | F: atgaggaaagtgaaaagatg; R: cagttctgattttttctactaa |
| rsv_circ_969 | F: ctgcaatgttgacatattcaacc; R: gcatgagcaactgcaagttttg |
| rsv_circ_305 | F: tccccactttatagaggtagc; R: tcctaatcacggctgtaagac |
| rsv_circ_443 | F: gatgttatagggctttctttgg; R: gaacaagcccaatggaaaag |
| rsv_circ_526 | F: caaagaaaacctcttccatg; R: ctattgagttgacagatatg |
| rsv_circ_561 | F: aacctctagtaagttgatgc; R: ctattgagttgacagatatg |
| rsv_circ_443  (Specific RT Primer) | GAACAAGCCCAATGGAAAAG |
| rsv_circ_969  (Specific RT Primer) | GCATGAGCAACTGCAAGTTTTG |
| siRSC1 | 1#: CTCAAAGAAGAAAGGTAAA  2#: AGAAGAAAGGTAAAGTGTT  3#: GAAGAAAGGTAAAGTGTTG |
| siRSC3 | 1#: GTTATGCTGAGAGATGTTA  2#: GATCGTTATGCTGAGAGAT |
| siRSC4 | 1#: CCAGACTGTTCTGTAGGAA  2#: AGGGCCAGACTGTTCTGTA  3#: GCCAGACTGTTCTGTAGGA |
| siRSC5 | 1#: TCCTGATTTGGGAAAATAA  2#: GGAAAATAACCACCTGCCT  3#: TGATTTGGGAAAATAACCA |
| siRSC6 | 1#: TGATGAAGAAAGATGTTCA  3#: CTGTGATGAAGAAAGATGT |
| siRSC7 | 1#: ATCGTGATAAAGCGGACAA  2#: GATCGTGATAAAGCGGACA  3#: GATAAAGCGGACAAGGCAA |
| siRSC8 | 1#: ACAAAACTGCTCCAGAAGC  2#: CAAAACTGCTCCAGAAGCC |
| siRSC9 | 1#: CCCAAAACTGTTGTCAGAA  2#: CAAAACTGTTGTCAGAAAA  3#: GTTGTCAGAAAAAGTGGAC |
| siRSC10 | 1#: CCAGAGGAATTTATGCAAA  2#: GCCCAGAGGAATTTATGCA  3#: CAGAGGAATTTATGCAAAT |
| ALN-RSV01 | GGCUCUUAGCAAAGUCAAGdTdT |

**Supplementary Table 1B** TFBS prediction using AnimalTFDB3.0 and analysis of potential ORFs and IRESs using circRNADb and CSCD

| **Alias Name** | **Best transcript** | **IFN-related TF by AnimalTFDB3.0**  **(TFBS_num)** | **circRNADb** | | **CSCD**  **(ORF)** |
| --- | --- | --- | --- | --- | --- |
|  |  |  | **IRES ORF** | |  |
| RSC1 | [NM_020183](http://www.ncbi.nlm.nih.gov/nuccore/NM_020183)/  ENST00000266503.9 | IRF3(22), STAT1(63), STAT2(14), RELA(110) | Yes (2) | No | Yes (5) |
| RSC2 | [NM_020183](http://www.ncbi.nlm.nih.gov/nuccore/NM_020183)/  ENST00000266503.9 | IRF3(22), STAT1(63), STAT2(14), RELA(110) | Yes (2) | No | Yes (1) |
| RSC3 | [NM_001024465](http://www.ncbi.nlm.nih.gov/nuccore/NM_001024465)/  ENST00000367055.8 | IRF3(37), STAT1(54), STAT2(17), RELA(105) | Yes (2) | Yes (149 aa) | Yes (1) |
| RSC4 | [NM_020119](http://www.ncbi.nlm.nih.gov/nuccore/NM_020119)/  ENST00000242351.9 | IRF3(15), STAT1(89), STAT2(29), RELA(34) | No records | No records | Yes (3) |
| RSC5 | [NM_016323](http://www.ncbi.nlm.nih.gov/nuccore/NM_016323)/  ENST00000264350.7 | IRF3(49), STAT1(69), STAT2(24), RELA(53) | Yes (2) | Yes (318 aa) | Yes (5) |
| RSC6 | [NM_001080391](http://www.ncbi.nlm.nih.gov/nuccore/NM_001080391)/  ENST00000340126.8 | IRF3(44), STAT1(104), STAT2(24), RELA(205) | Yes (2) | Yes (245 aa) | No |
| RSC7 | [NM_138426](http://www.ncbi.nlm.nih.gov/nuccore/NM_138426)/  ENST00000223145.9 | IRF3(27), STAT1(46), STAT2(13), RELA(27) | Yes (2) | No | Yes (16) |
| RSC8 | [NM_007203](http://www.ncbi.nlm.nih.gov/nuccore/NM_007203)/  ENST00000374530.7 | IRF3(24), STAT1(39), STAT2(17), RELA(120) | Yes (2) | Yes (825 aa) | Yes (1) |
| RSC9 | [NM_001008211](http://www.ncbi.nlm.nih.gov/nuccore/NM_001008211)/  [ENST00000378748.7](http://uswest.ensembl.org/Homo_sapiens/Transcript/Summary?db=core;g=ENSG00000123240;r=10:13099449-13138308;t=ENST00000378748) | IRF3(166), STAT1(86), STAT2(22), RELA(148) | Yes (2) | Yes (134 aa) | Yes (4) |
| RSC10 | [NM_020119](http://www.ncbi.nlm.nih.gov/nuccore/NM_020119)/  ENST00000242351.9 | IRF3(15), STAT1(89), STAT2(29), RELA(34) | Yes (2) | Yes (105 aa) | No |

TF, transcription factor; TFBS, TF binding site; TFBS_num, the number of TFBS; RELA, the p65 protein of NF-κB.
