# Supplementary material for: The Cellular and Viral circRNAome Induced by Respiratory Syncytial Virus Infection
Source: mBio. 2021 Dec 7;12(6):e03075-21. doi: 10.1128/mBio.03075-21 (PMC8649777; doi:10.1128/mBio.03075-21)
Supplement: TEXT S1 [file mbio.03075-21-s0001.docx]

**Supplementary Materials and methods**

***AGO2-binding sites from CLIP data sets.***

The evidence for AGO2-binding sites was obtained from a curated online cross-linking immunoprecipitation (CLIP) database doRiNA (http://dorina.mdc-berlin.de). These data sets include AGO2 PAR-CLIP and HITS-CLIP data from HEK-293 cells and several lymphoid cell lines. We downloaded the available data sets (http://dorina.mdc-berlin.de/regulators) and acquired the AGO2-binding sites of circRNA genomic region.

***TFBS prediction***

Sequence regions were retrieved at promoter/upstream of best mRNA transcript by 2000 bases using Table Browser of UCSC, and then transcriptional factor binding site (TFBS) prediction was conducted using AnimalTFDB 3.0 Tools (1) with these sequence regions.

***ORF and IRES prediction***

Potential ORFs of circRNAs were predicted with two web databases cancer-specific circRNA database (CSCD) (2) and circRNADb (3). Potential IRESs of circRNAs were predicted with circRNADb (3).

**Reference:**

1. Hu H, Miao YR, Jia LH, Yu QY, Zhang Q, Guo AY. 2019. AnimalTFDB 3.0: a comprehensive resource for annotation and prediction of animal transcription factors. Nucleic Acids Res 47:D33-D38.

2. Xia S, Feng J, Chen K, Ma Y, Gong J, Cai F, Jin Y, Gao Y, Xia L, Chang H, Wei L, Han L, He C. 2018. CSCD: a database for cancer-specific circular RNAs. Nucleic Acids Res 46:D925-D929.

3. Chen X, Han P, Zhou T, Guo X, Song X, Li Y. 2016. circRNADb: A comprehensive database for human circular RNAs with protein-coding annotations. Sci Rep 6:34985.
